# Beyond Essential Oils: Diterpenes, Lignans, and Biflavonoids from *Juniperus communis* L. as a Source of Multi-Target Lead Compounds

**DOI:** 10.3390/plants13223233

**Published:** 2024-11-17

**Authors:** Alina Arabela Jojić, Sergio Liga, Diana Uţu, Graţiana Ruse, Liana Suciu, Andrei Motoc, Codruța Marinela Şoica, Diana-Simona Tchiakpe-Antal

**Affiliations:** 1 Department of Pharmacology-Pharmacotherapy, Faculty of Pharmacy, “Victor Babes” University of Medicine and Pharmacy Timisoara, 2nd Eftimie Murgu Square, 300041 Timisoara, Romania; alina.jojic@umft.ro (A.A.J.); sergio.liga96@gmail.com (S.L.); suciu.liana@umft.ro (L.S.); codrutasoica@umft.ro (C.M.Ş.); 2 Research Center for Pharmacotoxicologic Evaluations (FARMTOX), “Victor Babes” University of Medicine and Pharmacy Timisoara, 2nd Eftimie Murgu Square, 300041 Timisoara, Romania; diana.antal@umft.ro; 3 Department of Applied Chemistry and Engineering of Organic and Natural Compounds, Faculty of Chemical Engineering, Biotechnologies and Environmental Protection, Politehnica University Timisoara, 6 Vasile Parvan, 300223 Timisoara, Romania; 4 Department of Pharmaceutical Botany, Faculty of Pharmacy, “Victor Babes” University of Medicine and Pharmacy Timisoara, 2nd Eftimie Murgu Square, 300041 Timisoara, Romania; gratiana.ruse@umft.ro; 5 Department of Anatomy-Embryology, Faculty of Medicine, “Victor Babes” University of Medicine and Pharmacy Timisoara, 2nd Eftimie Murgu Square, 300041 Timisoara, Romania; amotoc@umft.ro

**Keywords:** *Juniperus communis*, non-volatile metabolites, totarol, ferruginol, sugiol, scaffold

## Abstract

Common Juniper (*Juniperus communis* L.) is a gymnosperm that stands out through its fleshy, spherical female cones, often termed simply “berries”. The cone berries and various vegetative parts (leaves, twigs and even roots) are used in traditional phytotherapy, based on the beneficial effects exerted by a variety of secondary metabolites. While the volatile compounds of *Juniperus communis* are known for their aromatic properties and have been well-researched for their antimicrobial effects, this review shifts focus to non-volatile secondary metabolites—specifically diterpenes, lignans, and biflavonoids. These compounds are of significant biomedical interest due to their notable pharmacological activities, including antioxidant, anti-inflammatory, antimicrobial, and anticancer effects. The aim of this review is to offer an up-to-date account of chemical composition of *Juniperus communis* and related species, with a primary emphasis on the bioactivities of diterpenes, lignans, and biflavonoids. By examining recent preclinical and clinical data, this work assesses the therapeutic potential of these metabolites and their mechanisms of action, underscoring their value in developing new therapeutic options. Additionally, this review addresses the pharmacological efficacy and possible therapeutic applications of *Juniperus communis* in treating various human diseases, thus supporting its potential role in evidence-based phytotherapy.

## 1. Introduction

Since ancient times plants have been used as remedies against human ailments according to documents, monuments, and even preserved original plant products [1]; starting with simple pharmaceutical formulations such as infusions and macerations, the use of medicinal plants changed dramatically once active phytochemicals were identified and extracted in the early 19th century [2]. Although the drying process of plants may induce changes in their phytochemical composition [3], studies showed that the use of total extracts usually trigger more complex and longer-lasting biologic activity compared to pure compounds [4].

Currently, most pharmacopoeias contain monographs describing drugs of plant origin with real therapeutic values; herbal pharmacopoeias have also been published [5]. Natural products provide around 50% of modern drugs [6]; in addition, the market of herbal medicines has developed explosively based on traditional medicine, making up an estimated of 425 million USD in 2022 [7]; the use of plant-based products often involves self-medication as such or in combination with synthetic drugs leading to beneficial outcomes but also potentially harmful side effects or pharmacological interactions.

In order to achieve an effective therapy, the identification of plant components in terms of pharmacological effects is just as important as the proper diagnosis of the patient [8]; therefore, rational (evidence-based) phytotherapy has developed consisting in the use of those products whose therapeutic use is based on scientific research that identified active ingredients and effective dosage in both preclinical and clinical settings.

*Juniperus* genus comprises plants that can be used as source for the cedarwood oil that is used in folk medicine as antimicrobial and antibiofilm agent [9]; the plants have been reported with antimicrobial [10], cytotoxic [11], diuretic [12], anti-inflammatory [13], analgesic [14], hepatoprotective [15], antidiabetic [16] and hypolipidemic [17] activity. The genus *Juniperus* comprises around 75 species widely distributed mainly in cold and temperate regions but also reaching tropical areas [12]; in Romania, Juniper-based products (infusion, tincture, decoct) exploit the fruits, bark and aerial parts as well as the whole plant in both internal and external applications.

The current review aims to describe the chemical composition of *J. communis* L., together with the pharmacological effects and therapeutic benefits of extracts and selected pure compounds pertaining to the diterpene, lignan and biflavonoid subclasses. Both preclinical and clinical data are presented, supporting their use in human therapies. For some of the presented compounds (totarol, ferruginol), a large body of experimental evidence accumulated that deserve to be summarized. For other compounds (imbricatolic acid, pimaric acid, sandaracopimaric acid), promising data on the bioactivity are emerging. On the other hand, there has been progress characterizing the bioactivity of Juniper extracts. Studying the properties that individual metabolites contained in these extracts have, enable us a to better understand the effects of Juniper extracts, but also to guide further, more meaningful research on this plant.

## 2. Botanical Aspects

*Juniperus communis*, also known as common juniper, is a small evergreen shrub or a small coniferous evergreen tree that belongs to the *Cupressaceae* family [11,18,19,20,21,22]. It is native to the northern hemisphere, and can be found in various habitats, such as forests, heathlands, and mountains [19,22,23]. Geographical distribution and morphological differences determine the classification of the species into several subspecies and varieties [19]. The Juniper plant grows to a height of 1–3 m and has a dense, conical crown. The bark is reddish-brown with a rough, scaly texture and tends to peel off in thin strips Its leaves are needle-like, sharp at the tip, grouped in whorls of three, with a vibrant green color [12,21].

The female reproductive organs are spherical, consisting of three layered carpel scales that contain eggs. The male reproductive organs are oval, yellow, and feature numerous stamens [21. In *Juniperus communis*, male and female cones typically grow on separate plants. The female cones are larger and require about 18 months to reach maturity. [18,21]. *Juniperus communis* cones are a unique feature and frequently employed as a flavoring agent in cooking and gin production. The pseudo-fruits are globose and short-stemmed [12,19].

For centuries, it has been popular to use the essential oil derived from the berries and needles of the Juniper plant due to its aromatic, and therapeutic properties [24,25]. The unique composition, which incorporates a variety of terpenes, gives it a refreshing, woody, and slightly fruity fragrance, which makes it a popular choice for aromatherapy, and perfumery [12,18].

## 3. General Chemical Composition of the Juniper Plant

**Figure 1:**
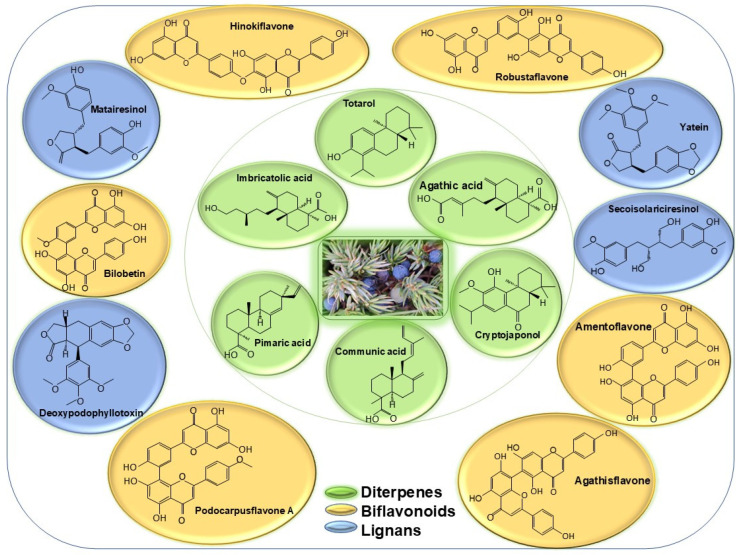
Main non-volatile secondary metabolites of *Juniperus communis* L.

The common Juniper plant is known to possess an extensive array of biologically active compounds, including: terpenes (volatile monoterpenes and non-volatile diterpenic derivatives), lignans, flavonoids, organic acids (glycolic, malic, and ascorbic), proteins, fermentable sugars, wax, and other compounds (Figure 1) [23,26,27]. The volatile compounds that make up the Juniper essential oil have received extensive attention [28,29,30,31]. They include monoterpene hydrocarbons (α-pinene, β-pinene, myrcene, sabinene, limonene), oxygenated monoterpene derivatives (terpinen-4-ol, borneol,), and sesquiterpenes (germacrene, *β*-caryophyllene) [28]. The content and composition of volatiles in Juniper cone berries varies widely from below 0.5% to over 3.5% [32]. In contrast, the current review focuses on the non-volatile secondary metabolites. This article aims to complement and update other excellent reviews on the topic of Juniper. Seca and Silva [33] made a comprehensive inventory of compounds published between 1970 and 2004 from various *Juniperus* species, continued by a later second review [34]. Gonçalves et al. [35] described the bioactivity of Juniper extracts, volatile organic compounds, phenolic acids, coumarins, anthocyanins, flavan-3-ols, proanthocyanidins, flavonols, flavones, carotenoids, and chlorophylls. Tavares and Seca [36] focused on a selection of diterpenes, flavonoids and one lignan. Other reviews [23,37] classified data according to the type of biologic activity exerted by various natural products from *Juniperus communis*.

The antimicrobial [10,38], antifungal [39,40,41], antioxidant [15], and anti-inflammatory [42,43] potentials of these compounds have been highlighted in several scientific articles up until now, along with their protective effects on various organs (liver, kidney) and against cancer [12,15,35,44]. Juniper plant extracts, essential oils, biologically active fractions, and individual compounds are viable options for the development of new molecules aimed at tackling acute and chronic human diseases. Our next section will consist of a short presentation on non-volatile bioactive compounds found in the Juniper plant.

### 3.1. Diterpenes

**Figure 2:**
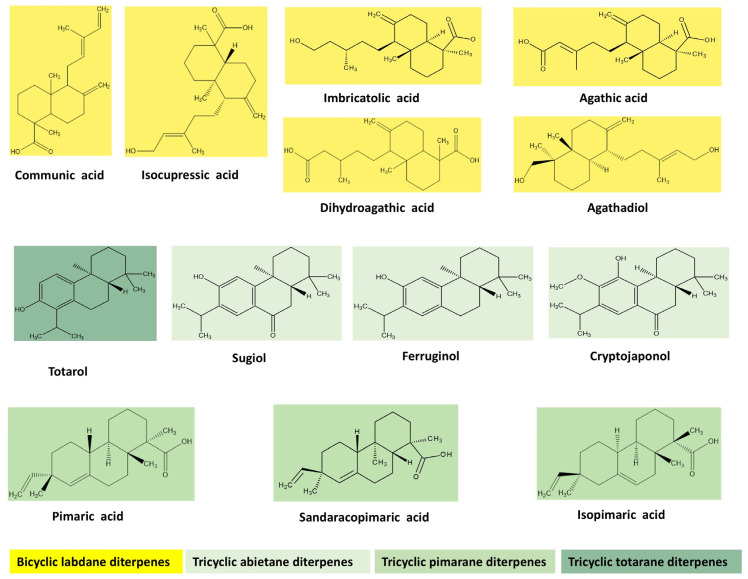
Prominent diterpenes found in *Juniperus communis* L.

Diterpenes are a variety of 20-carbon compounds that can be formed by condensing four isoprene units. Plants, animals, and fungi synthesize diterpenes through the 3-hydroxy-3-methyl-glutaryl-coenzymeA (HMG-CoA) reductase pathway, which involves the primary intermediate geranylgeranyl pyrophosphate [45,46,47]. The classification of diterpenes is determined by their core structures, and they can be classified as: (i) linear (phytane); (ii) monocyclic (retinol); (iii) bicyclic (clerodane, halimane, labdane); (iv) tricyclic (abietane, rosane, pimarane, podocarpane, cassane, vouacapane, chinane); (v) tetracyclic (kaurene, gibberellane, trachylobane, scopadulane, aphidicolane, atisane, stemodane, beyerene, stemarane); (vi) macrocyclic (cembrane, jatrophane, taxane, ingenane, daphnane, tigliane) [48,49,50]. Prominent diterpenes from Juniper pertaining to the above-mentioned classes are depicted in Figure 2.

Natural diterpenes and diterpenoids have been shown to have impressive biological activities, which could lead to their development in the pharmaceutical industry. In the next section, we will describe the most significant diterpenes found in juniper.

#### 3.1.1. Totarol

Totarol, also known as b,8,8-trimethyl-1-propan-2-yl-5,6,7,8a,9,10-hexahydrophenanthren-2-ol, is found in a variety of plants, such as *Podocarpus* and *Juniperus* spp., which are known to have bioactive properties [51,52]. It has been noted in *J. communis* roots [53].

Totarol has been shown to exhibit antibacterial activity against a wide range of bacteria, including both Gram-positive and Gram-negative bacteria. It disrupts bacterial cell membranes and inhibits bacterial growth, making it effective against various types of bacteria, including those responsible for common infections [54]

Harkenthal and co-workers investigated the antibacterial activity of totarol against a range of bacteria, including *Staphylococcus aureus*, *Escherichia coli*, and *Pseudomonas aeruginosa*. The researchers found that totarol was highly effective against these bacteria, with minimum inhibitory concentrations (MICs) ranging from 1–8 μg/mL [55]

Another study has concentrated on molecular targets and the mechanism of action of totarol in *Bacillus subtilis*. Their quantitative analysis of proteomes showed that diterpene causes changes in 139 protein expression levels. The same study also reports that the main central metabolic dehydrogenases of *Bacillus subtilis* are suppressed by totarol at the IC_50_ = 1.5 µM, leading to metabolic arrest in bacteria. [56]

Study conducted by Gordien and co-workers showed that totarol has the best activity against *Mycobacterium tuberculosis* genotype H37 Rv (MIC of 73.7 µM) and it was also most active against the isoniazid, streptomycin and moxifloxacin-resistant variants (MIC of 38.4, 83.4 and 60 µM) [53].

Several studies have demonstrated the antifungal activity of totarol. For instance, a study by Han and co-workers evaluated the antifungal activity of totarol against various clinical isolates of *Candida albicans* and found that it exhibited potent activity, with MIC values ranging from 4 to 32 µg/mL [57].

Totarol has demonstrated neuroprotective effects in both in vitro and in vivo models by safeguarding neurons from glutamate-induced cell death and oxygen-glucose deprivation. This protection is attributed to activation of the Akt/GSK-3β pathway, which boosts phosphorylation of Akt (protein kinase B) and GSK-3β (glycogen synthase kinase 3β), and increases Nrf2 (nuclear factor erythroid 2-related factor 2) and HO-1 (Heme oxygenase 1) protein expression, reducing oxidative stress. Totarol also enhances antioxidant defences by raising levels of glutathione (GSH) and superoxide dismutase (SOD), key in protecting cells from oxidative damage [58].

#### 3.1.2. Ferruginol

Abietanes, also known as abietane diterpenes, display a specific structure containing three fused six-membered rings and alkyl functional groups at C_4_, C_10_, and C_13_. The most relevant examples of abietanes are ferruginol and sugiol [59].

Ferruginol, or (4bS,8aS)-4b,8,8-trimethyl-2-propan-2-yl-5,6,7,8a,9,10-hexahydrophenanthren-3-ol, is a natural diterpene discovered in various parts of 31 species, with the majority of them belonging to the *Cupressaceae*, *Lamiaceae*, *Podocarpaceae*, and other minor families (e.g., *Taxaceae*, *Meliaceae*, *Martyniaceae*, *Pedaliaceae*, *Lauraceae*). The bark and root of species are the primary sources of ferruginol [59,60]. The compound has first been identified in *J. communis* in 1995 [61].

In the study by Thamaraiselvan and co-workers, ferruginol showed anti-inflammatory activity in MCF-7 breast cancer cells, effectively reducing inflammatory markers like TNF-α and IL-6. The IC50 for ferruginol’s anti-inflammatory effect in this model was determined to be 12 µM, highlighting its potential in modulating inflammation in cancer-related settings [62].

Ferruginol demonstrated a notable gastroprotective effect, reducing gastric lesions by 60% at 25 mg/kg, similar to lansoprazole. It also showed significant ulcer healing at 50 mg/kg with a 92.5% curative rate. Additionally, ferruginol inhibited lipid peroxidation with an IC50 of 1.4 µM and promoted cell proliferation, supporting its potential as an anti-ulcer agent [63].

Another study noted ferruginol for its antimicrobial properties, showing significant activity against various pathogens. It achieved an IC50 of 7.4 μg/mL against *Staphylococcus aureus* and 13.5 μg/mL against *Pseudomonas aeruginosa*, indicating its potential as a candidate for new antimicrobial development [64].

In the study by Takei and co-workers ferruginol was found to promote the differentiation of dendritic cells from human monocytes and enhance the generation of interleukin-10 (IL-10)-producing regulatory T cells in vitro. While the focus is on its immunological effects, the findings suggest potential neuroprotective activity related to immune regulation in the nervous system [65].

#### 3.1.3. Sugiol

Sugiol, or (4aS,10aS)-6-hydroxy-1,1,4a-trimethyl-7-propan-2-yl-3,4,10,10a-tetrahydro-2H-phenanthren-9-one, has been reported in 26 species, and it is particularly concentrated in *Cupressaceae* and *Lamiaceae* families, as well as minor family members such as *Taxaceae*, *Cladoniaceae*, and *Podocarpaceae* [59,66]. It has been isolated from several Juniper species, including *J. communis* [33].

Sugiol, exhibits significant antioxidant activity, as evidenced by various studies. Specifically, it has been shown to have an IC_50_ value of 36.32 µM when evaluated using the 2,2-diphenyl-1-picrylhydrazyl free radical scavenging assay. This indicates that sugiol effectively neutralizes free radicals, thereby contributing to its overall antioxidant capabilities [67].

Sugiol demonstrates significant antiproliferative activity against various cancer cell lines, particularly MCF-7, HeLa, and HCT116, with an IC50 value of about 22.45 µM. This suggests its potential as a therapeutic agent for inhibiting cancer cell growth [59].

Also, sugiol demonstrates strong antioxidant properties with an IC_50_ value of 36.32 µM, indicating its effectiveness in scavenging free radicals [66].

#### 3.1.4. Pimaric Acid, Sandaracopimaric Acid and Isopimaric Acid

Pimaric acid, or (1R,4aR,4bS,7S,10aR)-7-ethenyl-1,4a,7-trimethyl-3,4,4b,5,6,9,10,10a-octahydro-2H-phenanthrene-1-carboxylic acid, which is similar to abietane, is a type of diterpene resin and plays a significant role in pine oleoresin [68].

Pimaric acid shows strong antibacterial activity against *Paenibacillus larvae*, a bacterium harmful to honeybee colonies. The MIC of 6.25 µg/mL and zone of inhibition of 10–14 mm in agar diffusion assays causes disruption of the bacterial cell membrane, causing cell leakage, which contributes to its antibacterial effects. [69]

The study by Ishida and co-workers found that pimaric acid reduces vasoconstriction in rat pulmonary arterial smooth muscle by activating BKCa channels (large conductance calcium-activated potassium channels) and inhibiting VDCCs (voltage-gated calcium channels). This action increases potassium currents and lowers calcium influx, leading to muscle relaxation and reduced vasoconstriction. Pimaric acid specifically countered high potassium and endothelin-1-induced vasoconstriction, highlighting its potential as a vasorelaxant for vascular tension-related conditions [70].

Pimaric acid was shown to inhibit matrix metalloproteinase 9 (MMP-9) production in TNF-α-stimulated human aortic smooth muscle cells. This suppression of MMP-9 reduced cell migration, a key factor in inflammation and vascular remodelling. The effect was achieved by downregulating the NF-κB and AP-1 pathways, both critical for regulating inflammation and cell migration genes [71].

Sandaracopimaric acid, or (1R,4aR,4bS,7R,10aR)-7-ethenyl-1,4a,7-trimethyl-3,4,4b,5,6,9,10,10a-octahydro-2H-phenanthrene-1-carboxylic acid, is a tricyclic diterpene resin acid found in the resin of *Callitris* species [72].

Sandaracopimaric acid was shown to significantly reduce the contraction of pulmonary arteries induced by phenylephrine, indicating its vasodilatory effects. This vasodilation may be mediated by the endothelial nitric oxide synthase (eNOS) path-way and involves increased nitric oxide production in endothelial cells, with the potential role of the PI3K/Akt signalling pathway [73].

Isopimaric acid, or pyrrole-3-carboxaldehyde, is a diterpenoid present in many organisms naturally, such as *Boesenbergia rotunda* and *Picea obovata* [72].

These compounds have been pointed out in numerous Juniper species, including *J. communis* [33].

Isopimaric acid demonstrated significant anticancer activity against breast cancer cell lines such as MDA-MB-231 and MCF-7. The study reported an IC50 value of 6.81 μM, indicating its potency in inhibiting cell proliferation [74]. The antibacterial properties of isopimaric acid extracted from Pinus nigra, highlight its effectiveness against multidrug-resistant and methicillin-resistant *Staphylococcus aureus* (MRSA). The minimum inhibitory concentrations (MIC) were found to be between 32 and 64 µg/mL. Interestingly, isopimaric acid did not enhance the activity of antibiotics or show reduced MIC when combined with the efflux pump inhibitor reserpine; in some cases, MIC even increased [75].

#### 3.1.5. Imbricatolic Acid

Imbricatolic acid, or (1S,4aR,5S,8aR)-5-[(3S)-5-hydroxy-3-methylpentyl]-1,4a-dimethyl-6-methylidene-3,4,5,7,8,8a-hexahydro-2H-naphthalene-1-carboxylic acid, is a diterpenoid found in the resin extracted from the large tree *Araucaria araucana* (Mol.) Koch. It was obtained from the mature cone berries during research of compounds from Juniper that control the progression of the cell cycle [76]. Imbricatolic acid, have shown significant gastroprotective effects in mice, reducing gastric lesions by up to 78% at doses of 100 mg/kg—comparable to the proton pump inhibitor lansoprazole. Additionally, these compounds demonstrated cytotoxic activity, with IC_50_ values of 52 μM for AGS cells and 72 μM for fibroblasts, surpassing lansoprazole’s efficacy in similar assays [77]. Imbricatolic acid, isolated from *Juniperus communis*, has been examined for its effects on cell cycle progression in human anaplastic lung cancer CaLu-6 cells, which lack p53. It was found to induce G1 cell cycle arrest by promoting the accumulation of cyclin-dependent kinase inhibitors while degrading cyclins A, D1, and E1. This mechanism effectively halted the progression of the cell cycle, although it did not trigger significant apoptosis in the treated cells [76,78]. Imbricatolic acid has shown cytotoxic effects when subjected to biotransformation by *Aspergillus niger* and *Rhizopus nigricans*. Metabolites like 1α-hydroxyimbricatolic acid demonstrated moderate cytotoxicity, with IC_50_ values of 307μM against AGS human gastric cells and 631 μM against fibroblast cells. In comparison, imbricatolic acid itself exhibited stronger cytotoxicity, with IC_50_ values of 134 μM for AGS cells and 280 μM for fibroblasts, suggesting enhanced activity in its native form [79].

#### 3.1.6. Agathadiol, Agathic Acid, Dihydroagathic Acid

Labdane-related diterpenes, also known as secondary metabolites, are abundantly present in fungi, insects, higher plants, and marine organisms, and they exhibit a wide range of biological activities due to their high structural diversity. A fused decalin system (C_1-10_), with a six-carbon side chain at C_9_, composes the basic core structure of labdane diterpenes, and there are generally four carbons added to the decalin system at C_4_, C_8_, and C_10_ [80,81]. In labdane diterpenes class, the most representative bioactive molecules are agathic acid, agathadiol and dihydroagathic acid. Agathadiol was isolated from common Juniper by Feliciano et al. [61], while dihydroagathic acid was pointed out in leaves by Basas-Jaumandreu and co-workers [82].

The study by Salamone and co-workers identified that agathadiol extracted from juniper berries, functions as a positive allosteric modulator of the CB1 cannabinoid receptor. This interaction enhances CB1 receptor activity, suggesting agathadiol’s potential use in therapeutic areas like neuroprotection and pain management [83].

#### 3.1.7. Isocupressic Acid

Isocupressic acid, also chemically known as (1S,4aR,5S,8aR)-5-[(E)-5-hydroxy-3-methylpent-3-enyl]-1,4a-dimethyl-6-methylidene-3,4,5,7,8,8a-hexahydro-2H-naphthalene-1-carboxylic acid, is a bioactive compound that is used for abortion, and can be found in the needles of Ponderosa pine (*Pinus ponderosa* L.) [84,85]. In *J. communis*, isocupressic acid was proved in leaves [82].

In the study by Wu and co-workers isocupressic acid was shown to significantly inhibit progesterone production in bovine luteal cells by blocking luteinizing hormone (LH) activity, which is essential for progesterone synthesis [84].

#### 3.1.8. Cryptojaponol

Cryptojaponol, or (4aS,10aS)-5-hydroxy-6-methoxy-1,1,4a-trimethyl-7-propan-2-yl-3,4,10,10a-tetrahydro-2H-phenanthren-9-one, is a natural product found in *Juniperus formosana* and *Cryptomeria japonica* [86]. In the current plant, it is as well contained [87].

Cryptojaponol possesses cytotoxic properties, particularly against cancer cell lines, where it demonstrates moderate activity. For instance, in studies focusing on pancreatic cancer cells (PANC-1), cryptojaponol was reported to have an EC50 value of approximately 37.9 µM, indicating its potential as a chemotherapeutic agent [88]. Cryptojaponol has demonstrated activity against various bacterial strains, including *Staphylococcus aureus* and *Escherichia coli*. The compound was found to inhibit the growth of these bacteria at concentrations ranging from 50 µg/mL to 100 µg/mL, showing a potential for use in combating bacterial infections [89].

#### 3.1.9. Communic Acids

In *Cupresaceae* species, particularly in genus *Juniperus*, communic acids are a diterpenic natural product group with a labdane skeleton that contains three double bonds and a carboxyl group at C_19_ [90,91]. The most representative communic acids in common Juniper are trans-communic acid and cis-communic acid [33].

Communic acids, have shown strong antimicrobial activity against pathogens like *Staphylococcus aureus*, *S. epidermidis*, *Aspergillus fumigatus*, and *Candida albicans*. The reported LD50 in a brine shrimp bioassay is 0.16 μg/mL, indicating significant cytotoxic effects [90].

### 3.2. Lignans

**Figure 3:**
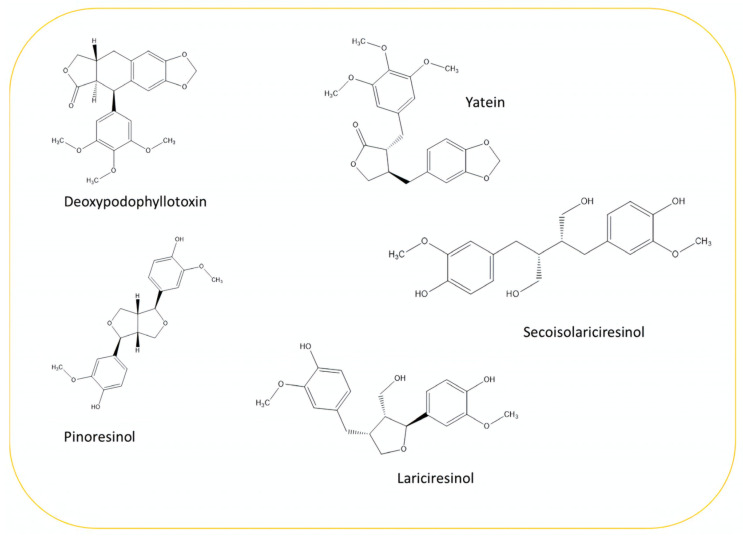
The main lignans found in *Juniperus communis* L.

Lignans are phenolic dimers with a 2,3-dibenzylbutane structure. It is known that these compounds are minor constituents in multiple plants, and act as building blocks for creating lignin in plant cell walls [92,93,94]. Most of the compounds are present in glycosidic form. Lignans can be divided into eight subgroups based on their cyclization pattern and oxygen incorporation into the skeleton, and each group includes: (i) furofuran; (ii) furan; (iii) dibenzylbutane; (iv) dibenzylbutyrolactone; (v) aryltetralin; (vi) arylnaphthalene; (vii) dibenzocyclooctadiene; and viii) dibenzobutyrolactol (Figure 3) [94,95]. Compound structures in every subgroup have significant differences in terms of the oxidation levels of both aromatic rings and propyl side chains [94].

#### 3.2.1. Deoxypodophyllotoxin and Podophyllotoxin

Among the lignans, cyclolignans present a carbohydrate cycle between the phenylpropane units, created by two single carbon-carbon bonds through the side chains [96,97]. Podophyllotoxin, which has a five-ring structure, is part of a class of compounds that includes several closely related chemical structures, such as podophyllotoxin, deoxypodophyllotoxin, 4′-demethylpodophyllotoxin, and α- and β-peltatins [96,98,99,100].

In *Juniperus communis*, the aryltetralin derivative deoxypodophyllotoxin is the main lignan [101]. Its content in leaves varies between 78 mg/100 g dry weight in ‘Horstman’ and 37.1 mg/100 g dry weight in ‘Gold cave’ varieties. Conversely, podophyllotoxin contents only span between 12.5 (‘Horstman’) and 2.2 mg/100 g dry weight (‘Depressa Aurea’). These compounds are of particular interest in antineoplastic therapies. The anticancer effects and mechanisms by which deoxypodophyllotoxin exerts them have recently been reviewed and based on research of numerous cell lines, including prostate cancer, cervical carcinoma, breast cancer, colorectal cancer, glioblastoma, oesophageal carcinoma [102]. Both mitochondrial and non-mitochondrial pathways are involved in the pro-apoptotic activity. Pharmacokinetic investigations performed in tumour-bearing mice showed that the compound had higher affinity for the cancer tissues than for plasma, and achieving particularly high concentrations in reproductive organs, fat and lungs [103]. Deoxypodophyllotoxin has as well other properties of medicinal interest including antibacterial, anti-inflammatory and antifertility [104,105,106,107,108,109,110,111,112,113,114,115,116,117,118,119,120,121,122,123,124,125,126,127,128,129,130,131,132,133,134,135,136,137,138,139,140,141,142,143,144,145,146,147,148,149,150,151,152,153,154,155,156,157,158,159,160,161,162,163,164] (Table 1).

Furthermore, in frontline cancer chemotherapy, two semisynthetic deoxypodophyllotoxin derivatives, etoposide and teniposide, are currently being used [105]. In addition to deoxypodophyllotoxin and podophyllotoxin, another derivative (β-peltatin-A-methylether) has been identified in the leaves of *J. communis* varieties ‘Laxa’ and ‘Oblonga pendula’ [106].

#### 3.2.2. Yatein

Yatein is a member of the class of butan-4-olides, or benzodioxoles, which contains 3,4,5-trimethoxybenzyl and 1,3-benzodioxol-5-yl-methyl substituents at positions 3 and 4. It is an important key biosynthetic intermediate of the antitumor lignan podophyllotoxin. The compound has so far been the subject of only a small number of promising investigations, mostly related to anticancer effects. Earlier research showed that compound has a strong inhibitory activity on the proliferation of murine myeloma [107]. The compound destabilizes microtubules and induces cell cycle arrest at the passage G_2_/M [108]. Recently, yatein was isolated during a bioactivity guided fractionation from *J. communis* cone berry water extract. It proved to be one of the active compounds that effectively suppressed the accumulation of lipofuscin in keratinocytes [109]. These findings should encourage further research on this lignan.

#### 3.2.3. Matairesinol

This compound has been identified in the leaves of common Juniper (‘Laxa’ and ‘Oblonga pendula’ varieties [106]. It is a constituent of the cuticular waxes that cover the leaves where it is the second most abundant lignan after deoxypodophyllotoxin, present in amounts of 18 mg/kg dry weight [82]. The compound has been intensively studied. It retained attention mostly due to its potential to fight cancer. It is able to inhibit histone deacetylase 8, an enzyme involved in the proliferation, invasion and metastasis of cancer cells [110]. The compound has been tested on various cell lines (prostate, breast, cervical, and pancreatic cancer); at least one of the mechanisms involves induction of mitochondrial impairment [111]. It synergized with 5-fluorouracil to combat pancreatic cancer [111] and is able to restore chemosensitivity in colorectal cancer cells [112].

Moreover, matairesinol combats oxidative damage and has anti-inflammatory properties. These abilities are involved in its reduction of pathologic cardiac remodelling [113], the improvement of experimentally induces brain injury [114] and suppression of neuroinflammation [115].

#### 3.2.4. Lariciresinol

This metabolite is, like matairesinol and yatein, a member of the dibenzylbutyrolactone lignan group. It is a minor compound found in the epicuticular wax of common Juniper leaves (2.9 mg/kg dry weight) [82]. It has been investigated in its capacity to combat diabetes via enhancement of insulin signalling and alpha-glucosidase inhibition [116], to counteract oxidative stress [117] and to fight pathogenic bacteria (*Staphylococcus aureus*, *Escherichia coli*) [118]. The compound was shown to act as an efflux pump inhibitor and combat drug resistant *Salmonella typhimurium* [119]. The anti-inflammatory properties of lariciresinol proved in a rat model of experimentally induced arthritis make the compound a promising candidate in the treatment of this condition [120]. The effect of this lignan against cancer showed inhibition of tumour growth and angiogenesis in rats bearing MCF-7 breast cancer xenografts [121] and apoptosis induction mediated through mitochondrial pathways in HepG2 cells [122].

#### 3.2.5. Secoisolariciresinol

This lignan, a derivative of 9,9′-dihydroxybenzylbutane, is constituents of leaf cuticles, where it amounts to about 12.9 mg/kg dry leaves [82]. Until the present, the compound as such (aglycone form) has only marginally been studied in comparison to its diglucoside present mainly in flaxseed, considered a major dietary phytoestrogen [123]. Chronic administration of equimolar amounts of secoisolariciresinol and its diglucoside had a similar activity profile, reducing weight gain, and lipid accumulation at hepatic level [124].

#### 3.2.6. Pinoresinol

Pinoresinol is a furanofurane type lignan, occurring in cuticular waxes of Juniper leaves (14.9 mg/kg dry weight) [82]. It displayed an intense fungicide effect against *Candida albicans*, damaging the plasma membrane of this pathogenic fungus [39]. With regard to an antibacterial effect, research showed the same site for a disruptive effect—the plasma membrane, leading to the leakage of soluble saccharides and proteins [125]. The compound has an anti-hyperglycaemic effect via inhibition of α-glucosidase [126]. Its anticancer activity in breast cancer cells was evaluated in HEK-293 and SkBr3 cell lines. The experimental setting included two other lignans, lariciresinol and podophyllotoxin. The values of half-maximal inhibitory concentrations, at 48h after application of pinoresinol, were 550 µM (HEK-293 cells) and 575 µM (SkBr3 cells), being in the same range as for lariciresinol (475 µM, and 500 µM, respectively, for the two cell lines) but showed a much lower activity than podophyllotoxin (IC_50_ values of 0.075 µM and 0.175 µM, respectively) [127]. Most research concerning pinoresinol was however performed on its diglucoside, with implications on a cardiovascular level [128], and showing hepatoprotective [129], neuroprotective [130] and anti-osteoporosis [131] effects. Pinoresinol is considered to be an enterolignan precursor and as such offering protection against certain cancer types and cardiovascular diseases after nutritional intake [132].

### 3.3. Biflavonoids

**Figure 4:**
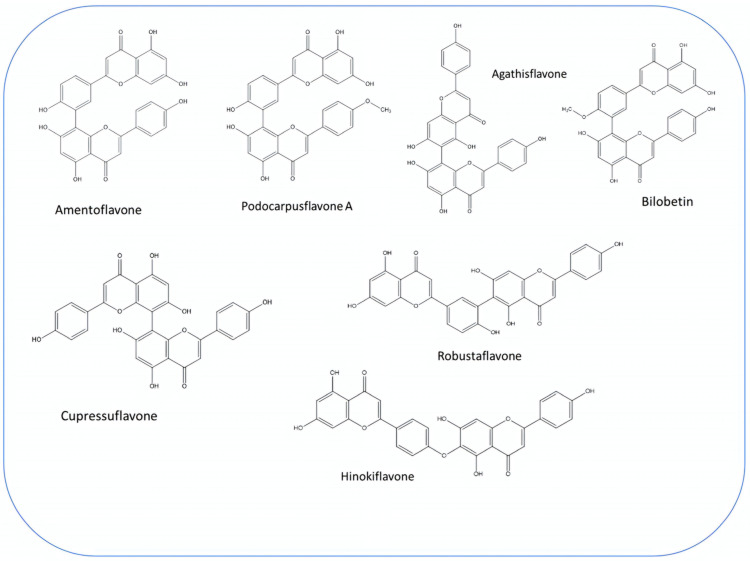
Main biflavonoids from *Juniperus communis* L.

Biflavonoids are composed of two monoflavonoid residues or flavonoids dimers. As summarized by He and co-workers, they occur naturally in angiosperms, bryophytes, and gymnosperms. Based on the presence or absence of an atom in the linker between the two residues, they can be classified into three groups (e.g., C-C linkages, C-linear fragments-C type, complex biflavonoids) (Figure 4) [133]. These compounds are present in many Juniper species, including common Juniper and contribute to some valuable bioactivities of these plants. Amentoflavone, hinokiflavone, cupressuflavone, and methyl-biflavones were isolated from *J. communis* cone berries in amounts ranging from 0.14 to 1.38 mg/g of fresh weight [134].

#### 3.3.1. Amentoflavone

Amenoflavone is an active polyphenolic compound, C_30_H_18_O_10_, classified as biflavonoid; while it is practically insoluble in water, it is easily soluble in alcohol due to its high hydrophobicity. In 1971, three plants from the *Selaginella* species and *Ginkgo biloba* were used as vegetal sources for its extraction for the first time. Since then, it has been discovered and extracted from more than 120 plants (e.g., *Amanoa almerindae*, *Alchornea glandulosa*, *Yersonima intermedia*, *Calophyllum pinetorum*, *Calophyllum membranaceum*, *Biota semipervirens*, *Antidesma bunius*, *Selaginella bryopteris*) [135]. In common Juniper it was discovered as early as 1975 [136], along with podocarpusflavone A and bilobetin. Its multifaceted therapeutic potential made it a subject of comprehensive reviews [137,138].

#### 3.3.2. Hinokiflavone

Hinokiflavone is a hydroxybiflavone with an aromatic ether structure that has been substituted at position 6 with a 4-(5,7-dihydroxy-4-oxo-4H-chromen-2-yl)-phenoxy group. It has been isolated from *Rhus succedanea*, *Metasequoia glyptostroboides*, *Garcinia multiflora*, and *Podocarpus elongatus* [139].

#### 3.3.3. Cupressuflavone

Cupressuflavone is a biflavonoid with the structural formula 8-[5,7-dihydroxy-2-(4-hydroxyphenyl)-4-oxochromen-8-yl]-5,7-dihydroxy-2-(4-hydroxyphenyl)-chromen-4-one that can be obtained by the oxidative coupling of two molecules of apigenin resulting in a bond between the two C_8_ positions of the chromene rings [140]. The isolation was achieved from *Cupressus sempervirens* and *Juniperus occidentalis* and isolated from several authors from *J. communis* [33].

#### 3.3.4. Podocarpusflavone A

Podocarpusflavone A is considered a natural compound that comes from the *Podocarpaceae* family and is classified as a cyclic nucleotide phosphodiesterase inhibitor [141]. Recently, this compound was isolated from the ethyl acetate fraction of cone berry extracts showing tyrosinase inhibition [142].

#### 3.3.5. Bilobetin

Bilobetin is a flavonoid oligomer acting as a potent inhibitor of the virus polymerase acidic endonuclease [143]. It effectively inhibits mutant PAN-I38T, and also displays anticancer activity against human hepatocellular carcinoma Huh7 and HepG2 cells [143,144].

#### 3.3.6. Agathisflavone

Agathisflavone, 8-[5,7-dihydroxy-2-(4-hydroxyphenyl)-4-oxochromen-6-yl]-5,7-dihydroxy-2-(4-hydroxyphenyl)-chromen-4-one, is a dimer of flavonoid apigenin that has been gaining attention due to its varied biological activities [145,146].

#### 3.3.7. Robustaflavone

By oxidating two molecules of apigenin one can obtain robustaflavone which contains both hydroxyphenyl and chromene rings; it has been reported to be isolated from *Thuja orientalis*, *Rhus succedanea*, *Schrebera trichoclada*, and *Nandina domestica* [147]. The compound was pointed out in common Juniper by [148].

In Table 1, we summarize some of the most important biological activities of the main bioactive compounds found in the juniper plant.

## 4. Recent Data on the Bioactivity of *Juniperus communis* Extracts

Many parts of *Juniperus communis* have been studied extensively following the extraction of their main ingredients; juniper’s medicinal and food preservation effects are attributed to its chemical constituents. Initially, research and marketing focused on using the plant for fragrance and flavouring, followed by its current medicinal uses based on traditional practices. The exploitation of its biologic effects, especially in the food, cosmetic, and health industries, is a result of recent research trends. As part of our review, we will analyse the biological activities of Juniper using recent data.

### 4.1. Antioxidant Activity

The antioxidant properties of *Juniperus* species are well documented and occur due to their rich phytochemical composition. Flavonoids, phenolic acids, and terpenes, which were described in previous paragraphs, are among the bioactive compounds found in such species that contribute to their antioxidant ability. *Juniperus* extracts have been tested for their antioxidant potential using different methods (e.g., DPPH radical-scavenging assays, Trolox equivalent antioxidant capacity assays, ferric reducing antioxidant power assays) to assess their effectiveness in scavenging free radicals, and preventing oxidative processes [165,166]. For example, in a study conducted by Höferl and co-workers it was shown that Juniper essential oil induces several mechanisms that facilitate radical scavenging, prevent radical formation (chelating capacity, inhibitory effect on xanthine oxidase) and protect against lipid peroxidation. The oil’s effects have also been proven in vivo where it blocked the oxidation processes in yeast cells and improved their adaptability to reactive oxidative species [27,167]. In the review on *Juniperus* species by Majid and co-workers, several studies on antioxidant activity were covered, noting that the high phenolic content in *Juniperus* extracts contributes to their antioxidant effects. For example, the ethanolic extract of *Juniperus communis* demonstrated strong antioxidant activity with an IC_50_ of approximately 28.55 µg/mL in DPPH assays (2,2-diphenyl-1-picrylhydrazyl) [168].

The study by Belov and co-workers explores the antioxidant activities of extracts from *Juniperus communis* berries, which were obtained using various solvents and extraction methods. Key assays include the DPPH radical scavenging assay and the ABTS (2,2′-azino-bis(3-ethylbenzothiazoline-6-sulfonic acid). For the ethanol extract, particularly the one derived through maceration, an IC_50_ of approximately 18 µg/mL was reported, reflecting a strong antioxidant potential. The acetone extract, processed through ultrasound-assisted maceration, displayed an IC_50_ around 23 µg/mL, which also shows significant activity but slightly less effective than the ethanol extract under the same conditions [26].

### 4.2. Antimicrobial Activity

In a study conducted by Zheljazkov and co-workers it was shown that the essential oil from Juniper cone berries, *Juniper galbulid*, with different phytochemical compositions can be obtained by capturing fractions at different time points during the hydrodistillation process. This study found that the essential oils extracted from juniper were active against *S. enterica* and *Klebsiella pneumonia*, among the three tested Gram-negative bacterial strains, along with *Clostridium perfringens* and *Candida glabrata*. Also, their results showed that certain essential oil fractions of *J. communis* have significant antimicrobial activity which could be utilized in various new antiseptic products or other biomedical applications [168]. According to the findings of Dumitrescu and co-workers, juniper’s essential oil caused a gradual decrease in the bacterial density for all tested strains. However, unlike Gram-negative bacteria, Gram-positive pathogens showed a significantly higher sensitivity, with *Staphylococcus aureus* being the most affected [18,169]. Muftah and co-workers evaluated the in vitro bioactivity of the active ingredient across various antimicrobial magistral drug formulations and plant extracts commonly used in folk medicine, finding similar MIC values ranging from 16 to 32 μg/mL [170].

### 4.3. Anti-Inflammatory Activity

Han and co-workers investigated the effects of juniper essential oils containing alpha-pinene as primary component, in human dermal fibroblasts designed to imitate chronic inflammation and fibrosis. Their observations revealed that juniper essential oils had a significant inhibitory impact on the production of two proinflammatory chemokines, interferon-induced protein 10 (IP-10) and interferon-induced T-cell alpha-chemoattractant (I-TAC), and also induced a decrease in collagen I, collagen III, and PAI-I [43]. According to experimental findings by Dae-Seung and co-workers, the pinene extracted from juniper species exhibits anti-inflammatory effects by inhibiting the phosphorylation of MAPKs (ERK, JNK), and the NF-B signalling pathway [171]. In a study published by Darwish and co-workers, it was found that Juniper essential oil inhibited the formation of pro-inflammatory cytokines, such as interleukin (IL)-1 and tumour necrosis factor (TNF-α) [172]. Extracts of *J. communis* have demonstrated the ability to reduce levels of pro-inflammatory cytokines, such as TNF-α and interleukin-1 beta (IL-1β), which are often elevated during inflammatory responses. For instance, an aqueous extract showed significant inhibition of prostaglandin synthesis (55% inhibition at 0.2 mg/mL) and platelet-activating factor (PAF) exocytosis (78% inhibition at 0.25 mg/mL) [23].

A specific study focused on *J. communis* extract’s protective effects against lipopolysaccharide (LPS)-induced acute kidney injury. It found that the extract activates the adenosine monophosphate-activated protein kinase (AMPK) pathway, which plays a critical role in cellular energy homeostasis and inflammatory responses. This activation contributes to the downregulation of inflammatory markers and protects against oxidative stress [173].

### 4.4. Anti-Cancer Activity

Tsai and co-workers’ study analysed the possibility of using *Juniper communis* extract as a treatment for cancer, with tests being conducted on BALB/c nude mice with subcutaneous glioblastoma. After sixteen days of treatment results showed that the tumour volume had significantly decreased. Their research suggests that juniper extract is a safe herbal remedy able to inhibit angiogenesis thus representing a promising therapeutic option against high-grade gliomas [174]. Lee and co-workers revealed that *Juniper communis* can induce p53 phosphorylation, Rb dephosphorylation, and p21 activation, resulting in a change in the expression of essential cell cycle proteins and triggering G0/G1 cell cycle arrest. Based on their data, it was revealed that juniper is capable of inducing both intrinsic and extrinsic apoptotic pathways thus contributing to cell death in human gingival squamous cancer cells [149]. In another study, Lai and co-workers investigated the in vitro and in vivo anticancer effects of juniper extract in colorectal cancer (CRC) cells. In vivo studies revealed that juniper extract is more effective against CRC cells than normal cells thus displaying selective antitumor effects and can interact with 5-fluorouracil in a synergistic manner. Cell apoptosis is caused by both internal (Bax/Bcl-2/caspase-9) and external (FasL/Fas/caspase-8) mechanisms, and cell cycle arrest is also triggered by regulating p53/p21 and CDK4/cyclin D1. In vivo studies showed that the extract suppressed tumour growth by inhibiting cell proliferation and inducing apoptosis [175]. Huang and co-workers focused on investigating the anticancer effect and underlying mechanism of *J. communis* extract in hepatocellular carcinoma cells both in vitro and in vivo. According to their results, juniper extract inhibited the expression of VEGF/VEGFR protein in vivo, similar to the findings reported in vitro, thus suggesting its ability to inhibit the autocrine and paracrine signalling pathways. Furthermore, juniper extract inhibited the formation of blood vessels and suppressed the MMP2/MMP9 protein expression in vivo, indicating that the extract has antimetastatic potential [44,176].

## 5. Future Perspectives

Studies on *Juniperus communis* showed the presence of valuable secondary metabolites such as diterpenes, lignans and biflavonoids that revealed a plethora of biological effects including antidiabetic, anti-bacterial, antioxidant and anticancer; hence, this species could be a reliable source of active compounds and standardized extracts that may provide therapeutic alternatives for a number of pathologies. As is the case with other phytocompounds, Juniperus phytochemicals may display poor pharmacokinetic profiles which makes them candidates for technological manipulation such as nanoformulations able to optimize their potential as therapeutic tools. To the best of our knowledge, the nanoformulation of *Juniperus communis* extracts has not been performed yet. Other Juniper species like *Juniperus procera* [177] and *Juniperus excelsa* [178,179] have been used to prepare nanoparticles, with the main field of utilization being antibacterial products such as wound dressings [178]. This type of application is meaningful taking into account both the antimicrobial effects of Juniper metabolites pointed out by modern research (and reviewed in this paper) but also the traditional indications of the plant [35]. Another field of interest is the study of natural products from Juniper in the supportive treatment of cancer. At least for the lignan matairesinol, a synergistic effect with 5-fluorouracil has been observed [111]. Furthermore, Juniper compounds contribute with their own cytotoxic effects, and have potent antioxidant and protective properties. In terms of toxicity, Juniper products was reported safe after short-term oral, inhalatory or topic administration; however, its long-term use especially in high doses may cause nephro- and gastrointestinal toxicity. Given the scarcity of toxicological studies published so far future tests should be conducted in order to clearly establish the extent and severity of adverse effects associated with the use of Juniper products via various administration routes. Despite the numerous biologic effects reported for Juniper phytocompounds clinical data in human subjects substantiating such therapeutic effects are still lacking therefore requiring future investigations. Preparation of extracts that are standardized in the main types of active principles (diterpenes, lignans, flavonoids) should be a prerequisite for effective Juniper-based pharmaceutical products.

## Figures and Tables

**Table 1:** The main bioactive compounds from juniper plant and their biological activities.

Class	Chemical Compound	Biological Activity
**Diterpenes**	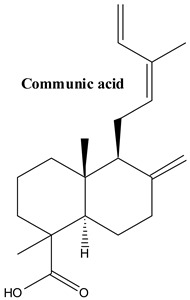	▪ Significant antibacterial and antifungal activity [90]
	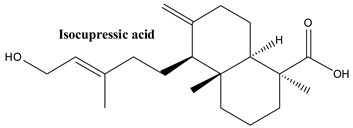	▪ Anti-mycobacterial activity; Inhibits transcription of P450scc, translation of StAR and P450scc through attenuating cAMP-PKA signaling [84]
	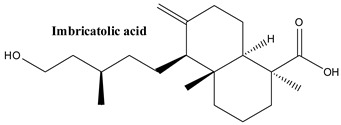	▪ Induces cell cycle arrest in CaLu-6 cells, by two possible mechanisms: (i) the accumulation of p21Cip1/Waf1/Sdi1, mediated by PKCδ activation and ERKs phosphorylation; and (ii) the decrease of cyclins A, D1, and E1 levels [76,78]
	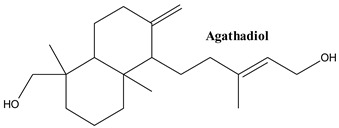	▪ Novel chemotype of positive allosteric modulator of CB1_R_; ▪ Significant cytotoxicity on NUGC and HONE-1 cells [83]
	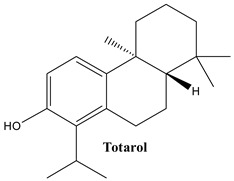	▪ Shows the potential for anti-inflammatory effects without exhibiting any cytotoxicity side effects [149] ▪ Exhibits anti-microbial activity against *Mycobacterium tuberculosis* H37 Rv (MIC = 73.7 M); also induced alterations in Bacillus subtilis proteome [56,150]; successfully inhibit Staphylococcus aureus, MICs for *S. aureus* strains were in the range of 2–4 lg/mL [54] ▪ Shows significant antibacterial effects against a wide range of bacteria towards oral bacteria and inhibition effects towards the development of the oral biofilm [151] ▪ Neuroprotective effects in primary neuronal cultures and in a brain ischemia rodent model, by activating the Akt/HO-1 pathway, thus contributing to a cellular anti-oxidative defense against neuronal injury [58]
	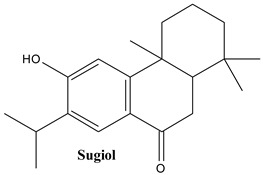	▪ Antiproliferative and apoptotic potential against human gastric cancer cells (SNU-5) [59] ▪ Induces activation of inflammatory signals via TL4-Myd88-MAPK kinase pathway [66] ▪ Active against promastigotes and amastigotes of *L. infantum* [152,153,154] ▪ Antioxidant, lipid peroxidation inhibitor [155]
	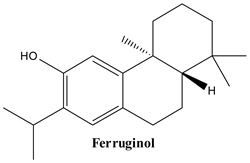	▪ Shows anticancer effects in MCF-7 cells [62] ▪ Gastroprotective effect by increasing the PGs content, protecting the cells against lipid peroxidation [63] ▪ Antimicrobial activity against *Staphylococcus aureus* including MRSA strains [64] ▪ Neuroprotective effect against MPTP-induced apoptosis and motor dysfunction [65]
	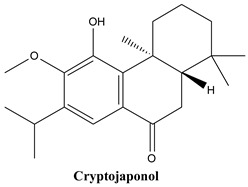	▪ Shows moderate cytotoxicity against PANC-1 cells under nutrient-starved conditions [155]
	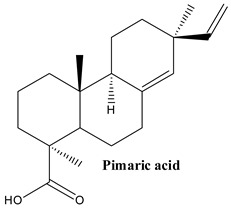	▪ Antibacterial activity against *Paenibacillus larvae* [69] ▪ Activates large-conductance Ca^2+^-activated K^+^ (BK_Ca_) channels and inhibit voltage-dependent Ca^2+^ channels (VDCCs) [70] ▪ Anti-atherosclerotic activity with inhibitory action on MMP-9 production and cell migration in TNF-alpha-induced HASMCs [71]
	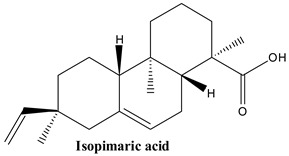	▪ Anti-tumor mechanism affects EMT and Wnt signaling pathways by targeting mitochondria oxidative phosphorylation and Ca^2+^ signaling pathways, and inducing breast cancer cell cycle arrest and apoptosis [74] ▪ Activity against Multidrug-resistant and EMRSA Strains of *Staphylococcus aureus* [75]
**Lignans**	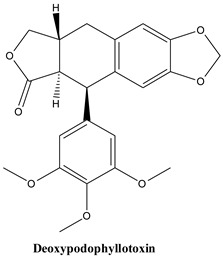	▪ Cytotoxic activity on a wide range of cancer cell lines; proapoptotic [102] ▪ Antibacterial activity; analgesic and anti-inflammation activities; antifertility effects [104]
	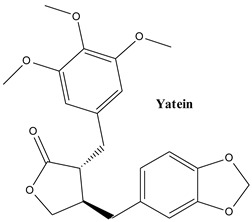	▪ Antiproliferative activity [107] ▪ Induces mitosis disturbance [108] ▪ Suppresses the accumulation of lipofuscin in keratinocytes [109]
	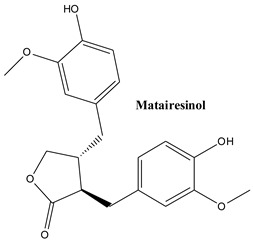	▪ Anticancer activity via inhibition of histone deacetylase 8 [110] ▪ Suppresses cell progression and migration, triggers apoptosis and mitochondrial dysfunction through MMP loss, and disturbed calcium regulation [111] ▪ Anti-inflammatory and antioxidant effects in sepsis-mediated brain injury by repressing the MAPK and NF-κB pathways through up-regulating AMPK [114]
	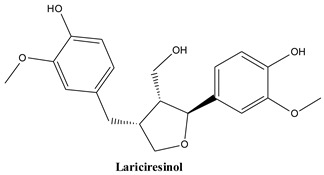	▪ Antidiabetic [116] ▪ Inhibits ROS generation in RAW 264.7 cells [117] ▪ Antibacterial activity [118] ▪ Combats drug resistance in bacteria [119] ▪ Anticancer [121,122]
	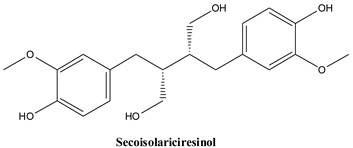	▪ Protective effects against cardiovascular diseases, diabetes, cancer, and mental stress; antioxidant activity [123] ▪ Reduction of weight gain [124]
	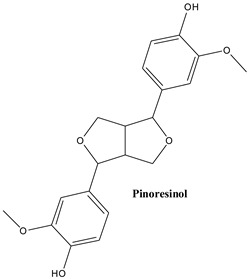	▪ Fungicide [39] ▪ Antibacterial activity [125] ▪ Anti-hyperglycemic activity [126] ▪ Weak cytotoxic effect [127] ▪ Hepatoprotective [129] ▪ Protective against cardiovascular diseases [132]
**Biflavones**	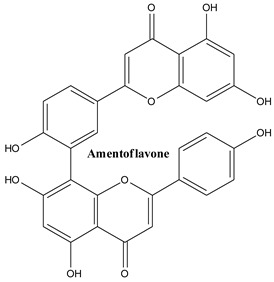	▪ Anti-inflammatory, anti-oxidation, antimicrobial activities [135] ▪ Antitumor activity; ▪ Antiviral activity; ▪ Anti-hypertrophic scar activity [156] ▪ Metabolism regulation, neuroprotection, musculoskeletal protection and antipsychotic effects [138,157]
	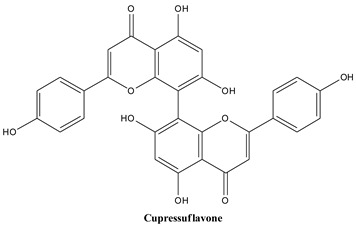	▪ Analgesic and anti-inflammatory effects [158] ▪ Antioxidant, antibacterial, antiviral activities [159]
	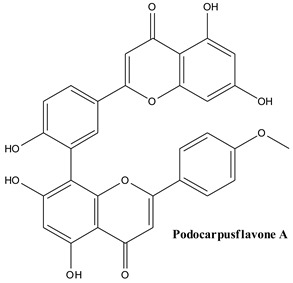	▪ Anti-inflammatory [160] ▪ Antiseptic, astringent, and antiproliferative activities [161]
	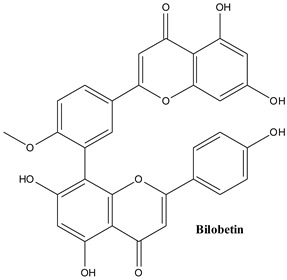	▪ Apoptotic effects and anticancer activity [144]
	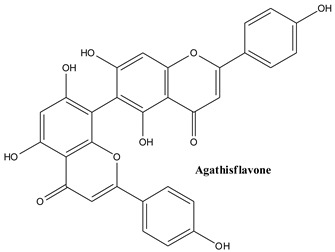	▪ Antiviral, antiparasitic, cytotoxic, neuroprotective, and hepatoprotective activities [145] ▪ Antioxidant, anti-inflammatory [146] ▪ SARS-CoV-2 replication inhibitor [162]
	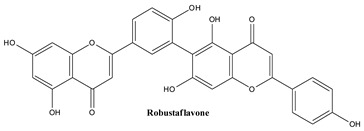	▪ Downregulates NO production in LPS-induced human colonic epithelial cells (HT-29) [147,163] ▪ Anti-angiogenic and pro-apoptotic effects [164]

## References

[1] Petrovska B.B. (2012). Historical Review of Medicinal Plants’ Usage. Pharmacogn. Rev..

[2] Sinha D., Odoh U.E., Ganguly S., Muhammad M., Chatterjee M., Chikeokwu I., Egbuna C., Egbuna C., Rudrapal M., Tijjani H. (2023). Chapter 1—Phytochemistry, History, and Progress in Drug Discovery. Phytochemistry, Computational Tools and Databases in Drug Discovery.

[3] Oliveira-Alves S.C., Andrade F., Prazeres I., Silva A.B., Capelo J., Duarte B., Caçador I., Coelho J., Serra A.T., Bronze M.R. (2021). Impact of Drying Processes on the Nutritional Composition, Volatile Profile, Phytochemical Content and Bioactivity of *Salicornia ramosissima* J. Woods. Antioxidants.

[4] Barba-Ostria C., Carrera-Pacheco S.E., Gonzalez-Pastor R., Heredia-Moya J., Mayorga-Ramos A., Rodríguez-Pólit C., Zúñiga-Miranda J., Arias-Almeida B., Guamán L.P. (2022). Evaluation of Biological Activity of Natural Compounds: Current Trends and Methods. Molecules.

[5] Kumar K.N.S. (2015). Herbal Pharmacopoeias– an Overview of International and Indian Representation. J. Ayurvedic Herb. Med..

[6] Anand U., Jacobo-Herrera N., Altemimi A., Lakhssassi N. (2019). A Comprehensive Review on Medicinal Plants as Antimicrobial Therapeutics: Potential Avenues of Biocompatible Drug Discovery. Metabolites.

[7] Bareetseng S. (2022). The Worldwide Herbal Market: Trends and Opportunities. J. Biomed. Res. Environ. Sci..

[8] Dzobo K., Kenakin T. (2022). The Role of Natural Products as Sources of Therapeutic Agents for Innovative Drug Discovery. Comprehensive Pharmacology.

[9] Kačániová M., Galovičová L., Valková V., Ďuranová H., Štefániková J., Čmiková N., Vukic M., Vukovic N.L., Kowalczewski P.Ł. (2022). Chemical Composition, Antioxidant, In Vitro and In Situ Antimicrobial, Antibiofilm, and Anti-Insect Activity of Cedar Atlantica Essential Oil. Plants.

[10] Khamis A.D.S., Chai L.C. (2021). Chemical and Antimicrobial Analyses of *Juniperus chinensis* and *Juniperus seravschanica* Essential Oils and Comparison with Their Methanolic Crude Extracts. Int. J. Anal. Chem..

[11] Ben Mrid R., Bouchmaa N., Bouargalne Y., Ramdan B., Karrouchi K., Kabach I., El Karbane M., Idir A., Zyad A., Nhiri M. (2019). Phytochemical Characterization, Antioxidant and In Vitro Cytotoxic Activity Evaluation of *Juniperus oxycedrus* Subsp. *oxycedrus* Needles and Berries. Molecules.

[12] Raina R., Verma P.K., Peshin R., Kour H. (2019). Potential of *Juniperus communis* L as a Nutraceutical in Human and Veterinary Medicine. Heliyon.

[13] Alkhedaide A.Q. (2018). Anti-Inflammatory Effect of *Juniperus procera* Extract in Rats Exposed to Streptozotocin Toxicity. Antiinflamm. Antiallergy Agents Med. Chem..

[14] Zhao J., Maitituersun A., Li C., Li Q., Xu F., Liu T. (2018). Evaluation on Analgesic and Anti-Inflammatory Activities of Total Flavonoids from *Juniperus sabina*. eCAM.

[15] Ved A., Gupta A., Rawat A. (2017). Antioxidant and Hepatoprotective Potential of Phenol-Rich Fraction of *Juniperus communis* Linn. Leaves. Pharmacogn. Mag..

[16] Orhan N., Watson R.R., Preedy V.R. (2019). Juniperus Species: Features, Profile, and Applications to Diabetes. Bioactive Food as Dietary Interventions for Diabetes.

[17] Kapadnis M.S., Pawar S., Dhikale R., Jadhav A. (2022). Studies on Several Medicinal Benefits of Plant *Juniperus communis*. Asian Pac. J. Health Sci..

[18] Dumitrescu E., Muselin F., Dumitrescu C.S., Orasan-Alic S.A., Moruzi R.F., Doma A.O., Mohamed E.A., Cristina R.T. (2022). *Juniper communis* L. Essential Oils from Western Romanian Carpathians: Bio-Structure and Effective Antibacterial Activity. Appl. Sci..

[19] Popescu D.I., Botoran O.R., Cristea R., Mihăescu C., Șuțan N.A. (2023). Effects of Geographical Area and Harvest Times on Chemical Composition and Antibacterial Activity of *Juniperus communis* L. Pseudo-Fruits Extracts: A Statistical Approach. Horticulturae.

[20] Jocienė L., Krokaitė E., Rekašius T., Vilčinskas R., Judžentienė A., Marozas V., Kupčinskienė E. (2023). Ionomic Parameters of Populations of Common *Juniperus communis* L. Depending on the Habitat Type. Plants.

[21] Salamon I., Otepka P., Kryvtsova M., Kolesnyk O., Hrytsyna M. (2023). Selected Biotopes of *Juniperus communis* L. in Slovakia and Their Chemotype Determination. Horticulturae.

[22] Thomas P.A., El-Barghathi M., Polwart A. (2007). Biological Flora of the British Isles: *Juniperus communis* L. J. Ecol..

[23] Bais S., Gill N.S., Rana N., Shandil S. (2014). A Phytopharmacological Review on a Medicinal Plant: *Juniperus communis*. Int. Sch. Res. Not..

[24] Tufail T., Ain H.B.U., Saeed A., Imran M., Basharat S., Nayik G.A., Nayik G.A., Ansari M.J. (2023). Chapter 17—*Juniper* Essential Oil: An Overview of Bioactive Compounds and Functional Aspects. Essential Oils.

[25] Bajac J., Zengin G., Mitrović I., Antić I., Radojković M., Nikolovski B., Terzić M. (2023). *Juniper* Berry Essential Oils as Natural Resources of Biological and Pharmacological High-Valuable Molecules. Ind. Crop. Prod..

[26] Belov T., Terenzhev D., Bushmeleva K.N., Davydova L., Burkin K., Fitsev I., Gatiyatullina A., Egorova A., Nikitin E. (2023). Comparative Analysis of Chemical Profile and Biological Activity of *Juniperus communis* L. Berry Extracts. Plants.

[27] Höferl M., Stoilova I., Schmidt E., Wanner J., Jirovetz L., Trifonova D., Krastev L., Krastanov A. (2014). Chemical Composition and Antioxidant Properties of *Juniper* Berry (*Juniperus communis* L.) Essential Oil. Antioxidants.

[28] Majewska E., Kozłowska M., Kowalska D., Gruczyńska E. (2017). Characterization of the Essential Oil from Cone-Berries of *Juniperus communis* L. (*Cupressaceae*). Herba Pol..

[29] Judžentienė A. (2019). *Juniperus communis* L.: A Review of Volatile Organic Compounds of Wild and Cultivated Common Juniper in Lithuania. Chemija.

[30] Albrecht U., Madisch A. (2022). Therapeutic Potentials Associated with Biological Properties of *Juniper* Berry Oil (*Juniperus communis* L.) and Its Therapeutic Use in Several Diseases—A Review. Bioact. Compd. Health Dis..

[31] Opruţa T., Tiţa M., Constantinescu A., Rusu L., Tiţa O. (2024). Characterization of *Juniperus communis* L. essential oil obtained from berries harvested from the Balkan area. Sci. Study Res. Chem. Chem. Eng. Biotechnol. Food Ind..

[32] Hajdari A., Mustafa B., Nebija D., Miftari E., Quave C.L., Novak J. (2015). Chemical composition of *Juniperus communis* L. cone essential oil and its variability among wild populations in Kosovo. Chem. Biodivers..

[33] Seca A.M.L., Silva A.M.S., Govil J.N., Singh V.K. (2006). The chemical composition of the *Juniperus* genus (1970–2004). Recent Progress in Medicinal Plants, Volume 16, Phytomedicines.

[34] Seca A.M., Pinto D.C., Silva A.M. (2015). The current status of bioactive metabolites from the genus *Juniperus*. Bioactive Phytochemicals: Perspectives for Modern Medicine.

[35] Gonçalves A.C., Flores-Félix J.D., Coutinho P., Alves G., Silva L.R. (2022). Zimbro (*Juniperus communis* L.) as a promising source of bioactive compounds and biomedical activities: A review on recent trends. Int. J. Mol. Sci..

[36] Tavares W.R., Seca A.M. (2018). The current status of the pharmaceutical potential of *Juniperus* L. metabolites. Medicines.

[37] Kakkar S., Bais S. (2014). A review on protocatechuic acid and its pharmacological potential. ISRN Pharmacol..

[38] Falcão S., Bacém I., Igrejas G., Rodrigues P.J., Vilas-Boas M., Amaral J.S. (2018). Chemical composition and antimicrobial activity of hydrodistilled oil from juniper berries. Ind. Crop. Prod..

[39] Hwang B., Lee J., Liu Q.H., Woo E.R., Lee D.G. (2010). Antifungal effect of (+)-pinoresinol isolated from *Sambucus williamsii*. Molecules.

[40] Cavaleiro C., Pinto E., Gonçalves M.J., Salgueiro L. (2006). Antifungal activity of *Juniperus* essential oils against dermatophyte, *Aspergillus*, and *Candida* strains. J. Appl. Microbiol..

[41] Filipowicz N., Kamiński M., Kurlenda J., Asztemborska M., Ochocka J.R. (2003). Antibacterial and antifungal activity of juniper berry oil and its selected components. Phytother. Res..

[42] Fierascu I., Ungureanu C., Avramescu S.M., Cimpeanu C., Georgescu M.I., Fierascu R.C., Ortan A., Sutan A.N., Anuta V., Zanfirescu A. (2018). Genoprotective, antioxidant, antifungal and anti-inflammatory evaluation of hydroalcoholic extract of wild-growing *Juniperus communis* L. (Cupressaceae) native to Romanian southern sub-Carpathian hills. BMC Complement. Altern. Med..

[43] Han X., Parker T.L. (2017). Anti-inflammatory activity of juniper (*Juniperus communis*) berry essential oil in human dermal fibroblasts. Cogent Med..

[44] Huang N.C., Huang R.L., Huang X.F., Chang K.F., Lee C.J., Hsiao C.Y., Lee S.C., Tsai N.M. (2021). Evaluation of anticancer effects of *Juniperus communis* extract on hepatocellular carcinoma cells in vitro and in vivo. Biosci. Rep..

[45] Hu Z., Liu X., Tian M., Ma Y., Jin B., Gao W., Cui G., Guo J., Huang L. (2021). Recent progress and new perspectives for diterpenoid biosynthesis in medicinal plants. Med. Res. Rev..

[46] Li H., Dickschat J.S. (2022). Diterpene biosynthesis from geranylgeranyl diphosphate analogues with changed reactivities expands skeletal diversity. Angew. Chem. Int. Ed..

[47] Liu Y., Chen X., Zhang C. (2023). Sustainable biosynthesis of valuable diterpenes in microbes. Eng. Microbiol..

[48] Ludwiczuk A., Skalicka-Woźniak K., Georgiev M.I. (2017). Terpenoids. Pharmacognosy.

[49] Khan R.A., Hossain R., Siyadatpanah A., Al-Khafaji K., Khalipha A.B.R., Dey D., Asha U.H., Biswas P., Saikat A.S.M., Chenari H.A. (2021). Diterpenes/diterpenoids and their derivatives as potential bioactive leads against dengue virus: A computational and network pharmacology study. Molecules.

[50] Toyomasu T., Sassa T., Liu H.-W., Mander L. (2010). Diterpenes. Comprehensive Natural Products II.

[51] Zhou L., Wang J., Wang K., Xu J., Zhao J., Shan T., Luo C., Atta-ur-Rahman (2012). Secondary metabolites with antinematodal activity from higher plants. Studies in Natural Products Chemistry.

[52] Bendall J.G., Cambie R.C. (1995). ChemInform Abstract: Totarol: A Non-Conventional Diterpenoid. ChemInform.

[53] Gordien A.Y., Gray A.I., Franzblau S.G., Seidel V. (2009). Antimycobacterial terpenoids from *Juniperus communis* L. (Cuppressaceae). J. Ethnopharmacol..

[54] Shi C., Che M., Zhang X., Liu Z., Meng R., Bu X., Ye H., Guo N. (2018). Antibacterial activity and mode of action of totarol against *Staphylococcus aureus* in carrot juice. J. Food Sci. Technol..

[55] Harkenthal M., Reichling J., Geiss H.K., Saller R. (1999). Comparative study on the in vitro antibacterial activity of Australian tea tree oil, cajuput oil, niaouli oil, manuka oil, kanuka oil, and eucalyptus oil. Pharmazie.

[56] Reddy P.J., Ray S., Sathe G.J., Gajbhiye A., Prasad T.S., Rapole S., Panda D., Srivastava S. (2015). A comprehensive proteomic analysis of totarol induced alterations in *Bacillus subtilis* by multipronged quantitative proteomics. J. Proteom..

[57] Han J., Li Q., Liu Y., Zhao L., Li X. (2015). Totarol: A natural antimicrobial and antifungal agent. J. Biosci. Bioeng..

[58] Gao Y., Xu X., Chang S., Wang Y., Xu Y., Ran S., Huang Z., Li P., Li J., Zhang L. (2015). Totarol prevents neuronal injury in vitro and ameliorates brain ischemic stroke: Potential roles of Akt activation and HO-1 induction. Toxicol. Appl. Pharmacol..

[59] Chan E.W.C., Wong S.K., Chan H.T. (2023). Ferruginol and sugiol: A short review of their chemistry, sources, contents, pharmacological properties, and patents. Trop. J. Nat. Prod. Res..

[60] Salih A.M., Al-Qurainy F., Tarroum M., Khan S., Nadeem M., Shaikhaldein H.O., Alansi S. (2022). Phytochemical compound profile and the estimation of the ferruginol compound in different parts (roots, leaves, and seeds) of *Juniperus procera*. Separations.

[61] Feliciano A.S., Medarde M., Gordaliza M., Lucas M.J. (1995). Structure elucidation of germacrane alcohols from *Juniperus communis* subsp. hemisphaerica. J. Nat. Prod..

[62] Thamaraiselvan R., Rengarajan S., Keerthiga S., Sivakumar D., Duraikannu P., Velu P., Velu P. (2018). Cancer+ Exploring the anticancer and anti-inflammatory activities of ferruginol in MCF-7 breast cancer cells. C+.

[63] Rodríguez J.A., Theoduloz C., Yáñez T., Becerra J., Schmeda-Hirschmann G. (2006). Gastroprotective and ulcer healing effect of ferruginol in mice and rats: Assessment of its mechanism of action using in vitro models. Life Sci..

[64] Olha A., Maranha A., Salvador J.A.R., Empadinhas N., Moreira V.M. (2024). Bi- and tricyclic diterpenoids: Landmarks from a decade (2013–2023) in search of leads against infectious diseases. Nat. Prod. Rep..

[65] Takei M., Umeyama A., Arihara S. (2005). Epicubenol and ferruginol induce DC from human monocytes and differentiate IL-10-producing regulatory T cells in vitro. Biochem. Biophys. Res. Commun..

[66] Bajpai V.K., Sonwal S., Hwang S.K., Shukla S., Khan I., Dey D.K., Chen L., Simal-Gandara J., Xiao J., Huh Y.S. (2021). Sugiol, a diterpenoid: Therapeutic actions and molecular pathways involved. Pharmacol. Res..

[67] Hao S., Meng Q., Sun H., Li Y., Li Y., Gu L., Liu B., Zhang Y., Zhou H., Xu Z. (2022). The role of transketolase in human cancer progression and therapy. Biomed. Pharmacother..

[68] Azemard C., Menager M., Vieillescazes C. (2016). Analysis of diterpenic compounds by GC-MS/MS: Contribution to the identification of main conifer resins. Anal. Bioanal. Chem..

[69] Song H., Kim J., Shin Y.K., Kim K.Y. (2022). Antibacterial activity of pimaric acid against the causative agent of American foulbrood, *Paenibacillus larvae*. J. Apic. Res..

[70] Ishida M., Yamamura A., Fujiwara M., Amano T., Ota M., Hikawa Y., Kondo R., Suzuki Y., Imaizumi Y., Yamamura H. (2023). Pimaric acid reduces vasoconstriction via BKCa channel activation and VDCC inhibition in rat pulmonary arterial smooth muscles. J. Pharmacol. Sci..

[71] Suh S.J., Kwak C.H., Chung T.W., Park S.J., Cheeeei M., Park S.S., Seo C.S., Son J.K., Chang Y.C., Park Y.G. (2012). Pimaric acid from *Aralia cordata* has an inhibitory effect on TNF-α-induced MMP-9 production and HASMC migration via down-regulated NF-κB and AP-1. Chem. Biol. Interact..

[72] Banerjee S., Manisha C., Murugan D., Justin A., El-Shemy H.A. (2021). Natural products altering GABAergic transmission. Natural Medicinal Plants.

[73] Gao W., Dong X., Xie N., Zhou C., Fan Y., Chen G., Wang Y., Wei T., Zhu D. (2014). Dehydroabietic acid isolated from *Commiphora opobalsamum* causes endothelium-dependent relaxation of pulmonary artery via PI3K/Akt-eNOS signaling pathway. Molecules.

[74] Li J., Liu X., Chen L., Zhu X., Yu Z., Dong L., Zhao X., Zou H., Wei Q., Feng Y. (2023). Isopimaric acid, an ion channel regulator, regulates calcium and oxidative phosphorylation pathways to inhibit breast cancer proliferation and metastasis. Toxicol. Appl. Pharmacol..

[75] Smith E., Williamson E., Zloh M., Gibbons S. (2005). Isopimaric acid from *Pinus nigra* shows activity against multidrug-resistant and EMRSA strains of *Staphylococcus aureus*. Phytother. Res..

[76] De Marino S., Cattaneo F., Festa C., Zollo F., Iaccio A., Ammendola R., Incollingo F., Iorizzi M. (2011). Imbricatolic acid from *Juniperus communis* L. prevents cell cycle progression in CaLu-6 cells. Planta Med..

[77] Frezza C., Venditti A., De Vita D., Toniolo C., Franceschin M., Ventrone A., Tomassini L., Foddai S., Guiso M., Nicoletti M. (2020). Phytochemistry, chemotaxonomy, and biological activities of the Araucariaceae family—A review. Plants.

[78] Woo K.W., Choi S.U., Park J.C., Lee K.R. (2011). A new lignan glycoside from Juniperus rigida. Arch. Pharm. Res..

[79] Schmeda-Hirschmann G., Aranda C., Kurina M., Rodríguez J.A., Theoduloz C. (2007). Biotransformations of Imbricatolic Acid by *Aspergillus niger* and *Rhizopus nigricans* Cultures. Molecules.

[80] Demetzos C., Dimas K.S., Atta-ur-Rahman (2001). Labdane-Type Diterpenes: Chemistry and Biological Activity. Studies in Natural Products Chemistry.

[81] Grant P.S., Brimble M.A. (2021). Seco-Labdanes: A Study of Terpenoid Structural Diversity Resulting from Biosynthetic C−C Bond Cleavage. Chem. Eur. J..

[82] Basas-Jaumandreu J., López J.F., de las Heras F.X. (2015). Labdane-type diterpenoids from *Juniperus communis* needles. Ind. Crop. Prod..

[83] Salamone S., Appendino G., Khalili A., Pollastro F., Munoz E., Unciti-Broceta J.D. (2021). Agathadiol, a Labdane Diterpenoid from Juniper Berries, Is a Positive Allosteric Modulator of CB1R. Fitoterapia.

[84] Wu L.S., Chen J.C., Sheu S.Y., Huang C.C., Kuo Y.H., Chiu C.H., Lian W.X., Yang C.J., Kaphle K., Lin J.H. (2002). Isocupressic Acid Blocks Progesterone Production from Bovine Luteal Cells. Am. J. Chin. Med..

[85] Stegelmeier B.L., Gardner D.R., James L.F., Panter K.E., Molyneux R.J. (1996). The Toxic and Abortifacient Effects of Ponderosa Pine. Vet. Pathol..

[86] Matsumoto T., Ohsuga Y., Harada S., Fukui K. (1977). Synthesis of Taxodione, Royleanone, Cryptojaponol, and Methyl 11-Hydroxy-12-Methoxy-7-Oxoabieta-8,11)13-Trien-18-Oate. Bull. Chem. Soc. Jpn..

[87] Schneider I., Gibbons S., Bucar F. (2004). Inhibitory activity of *Juniperus communis* on 12(S)-HETE production in human platelets. Planta Med..

[88] Kuźma Ł., Gomulski J. (2022). Biologically Active Diterpenoids in the *Clerodendrum* Genus—A Review. Int. J. Mol. Sci..

[89] González M.A. (2015). Aromatic abietane diterpenoids: Their biological activity and synthesis. Nat. Prod. Rep..

[90] Barrero A.F., Herrador M.M., Arteaga P., Arteaga J.F., Arteaga A.F. (2012). Communic Acids: Occurrence, Properties and Use as Chirons for the Synthesis of Bioactive Compounds. Molecules.

[91] Peters R.J. (2010). Two Rings in Them All: The Labdane-Related Diterpenoids. Nat. Prod. Rep..

[92] Osmakov D.I., Kalinovskii A.P., Belozerova O.A., Andreev Y.A., Kozlov S.A. (2022). Lignans as Pharmacological Agents in Disorders Related to Oxidative Stress and Inflammation: Chemical Synthesis Approaches and Biological Activities. Int. J. Mol. Sci..

[93] Yoder S.C., Lancaster S.M., Hullar M.A.J., Lampe J.W., Tuohy K., Del Rio D. (2015). Chapter 7—Gut Microbial Metabolism of Plant Lignans: Influence on Human Health. Diet-Microbe Interactions in the Gut.

[94] Simpson D., Amos S., Badal S., Delgoda R. (2017). Chapter 12—Other Plant Metabolites. Pharmacognosy.

[95] Gulcin İ. (2020). Antioxidants and Antioxidant Methods: An Updated Overview. Arch. Toxicol..

[96] Fan H., Zhu Z., Xian H., Wang H., Chen B., Tang Y.-J., Tang Y., Liang X. (2021). Insight into the Molecular Mechanism of Podophyllotoxin Derivatives as Anticancer Drugs. Front. Cell Dev. Biol..

[97] Cragg G.M., Pezzuto J.M. (2016). Natural Products as a Vital Source for the Discovery of Cancer Chemotherapeutic and Chemopreventive Agents. Med. Princ. Pract..

[98] Shah Z., Farooq Gohar U., Jamshed I., Mushtaq A., Mukhtar H., Zia-Ui-Haq M., Toma S.I., Manea R., Moga M., Popovici B. (2021). Biomolecules Podophyllotoxin: History, Recent Advances and Future Prospects. Biomolecules.

[99] Jin L., Song Z., Cai F., Ruan L., Jiang R. (2023). Chemistry and Biological Activities of Naturally Occurring and Structurally Modified Podophyllotoxins. Molecules.

[100] Miranda-Vera C., Hernández Á.P., García-García P., Díez D., García P.A., Castro M.Á. (2023). Podophyllotoxin: Recent Advances in the Development of Hybridization Strategies to Enhance Its Antitumoral Profile. Pharmaceutics.

[101] Och M., Och A., Cieśla Ł., Kubrak T., Pecio Ł., Stochmal A., Kocki J., Bogucka-Kocka A. (2015). Study of cytotoxic activity, podophyllotoxin, and deoxypodophyllotoxin content in selected *Juniperus* species cultivated in Poland. Pharm. Biol..

[102] Mottaghi S., Abbaszadeh H. (2022). A comprehensive insight into the antineoplastic activities and molecular mechanisms of deoxypodophyllotoxin: Recent trends, challenges, and future outlook. Eur. J. Pharmacol..

[103] Liu F., Zheng A., Li M., Chen Y., Liu X. (2024). Study on pharmacokinetics and tissue distribution of deoxypodophyllotoxin and its metabolites in tumour-bearing mice. Xenobiotica.

[104] Khaled M., Jiang Z.Z., Zhang L.Y. (2013). Deoxypodophyllotoxin: A promising therapeutic agent from herbal medicine. J. Ethnopharmacol..

[105] Clark P.I., Slevin M.L. (1987). The clinical pharmacology of etoposide and teniposide. Clin. Pharmacokinet..

[106] Ivanova D.I., Nedialkov P.T., Tashev A.N., Olech M., Nowak R., Ilieva Y.E., Kokanova-Nedialkova Z.K., Atanasova T.N., Angelov G., Najdenski H.M. (2021). Junipers of various origins as potential sources of the anticancer drug precursor podophyllotoxin. Molecules.

[107] Donoso-Fierro C., Tiezzi A., Ovidi E., Ceccarelli D., Triggiani D., Mastrogiovanni F., Taddei A.R., Pérez C., Becerra J., Silva M. (2015). Antiproliferative activity of yatein isolated from *Austrocedrus chilensis* against murine myeloma cells: Cytological studies and chemical investigations. Pharm. Biol..

[108] Ho S.T., Lin C.C., Tung Y.T., Wu J.H. (2019). Molecular mechanisms underlying yatein-induced cell-cycle arrest and microtubule destabilization in human lung adenocarcinoma cells. Cancers.

[109] Sakamoto K., Fujimoto R., Nakagawa S., Kamiyama E., Kanai K., Kawai Y., Kojima H., Hirasawa A., Wakamatsu K., Masutani T. (2023). Juniper berry extract containing anthricin and yatein suppresses lipofuscin accumulation in human epidermal keratinocytes through proteasome activation, increases brightness and decreases spots in human skin. Int. J. Cosmet. Sci..

[110] Mahajan M., Suryavanshi S., Bhowmick S., Alasmary F.A., Almutairi T.M., Islam M.A., Kaul-Ghanekar R. (2021). Matairesinol, an active constituent of HC9 polyherbal formulation, exhibits HDAC8 inhibitory and anticancer activity. Biophys. Chem..

[111] Lee W., Song G., Bae H. (2022). Matairesinol induces mitochondrial dysfunction and exerts synergistic anticancer effects with 5-fluorouracil in pancreatic cancer cells. Mar. Drugs.

[112] Wu S., Wang J., Fu Z., Familiari G., Relucenti M., Aschner M., Li X., Chen H., Chen R. (2023). Matairesinol nanoparticles restore chemosensitivity and suppress colorectal cancer progression in preclinical models: Role of lipid metabolism reprogramming. Nano Lett..

[113] Zhang T., Li L., Mo X., Xie S., Liu S., Zhao N., Zhang H., Chen S., Zeng X., Wang S. (2024). Matairesinol blunts adverse cardiac remodeling and heart failure induced by pressure overload by regulating Prdx1 and PI3K/AKT/FOXO1 signaling. Phytomedicine.

[114] Qin W., Wang Y., Li Q. (2021). Matairesinol exerts anti-inflammatory and antioxidant effects in sepsis-mediated brain injury by repressing the MAPK and NF-κB pathways through up-regulating AMPK. Aging.

[115] Xu P., Huang M.W., Xiao C.X., Long F., Wang Y., Liu S.Y., Jia W.W., Wu W.J., Yang D., Hu J.F. (2017). Matairesinol suppresses neuroinflammation and migration associated with Src and ERK1/2-NF-κB pathway in activating BV2 microglia. Neurochem. Res..

[116] Alam M.B., Ra J.S., Lim J.Y., Song B.R., Javed A., Lee S.H. (2022). Lariciresinol displays anti-diabetic activity through inhibition of α-glucosidase and activation and enhancement of insulin signaling. Mol. Nutr. Food Res..

[117] Bajpai V.K., Alam M.B., Quan K.T., Kwon K.R., Ju M.K., Choi H.J., Lee J.S., Yoon J.I., Majumder R., Rather I.A. (2017). Antioxidant efficacy and the upregulation of Nrf2-mediated HO-1 expression by (+)-lariciresinol, a lignan isolated from *Rubia philippinensis*, through the activation of p38. Sci. Rep..

[118] Bajpai V.K., Shukla S., Paek W.K., Lim J., Kumar P., Kumar P., Na M.K. (2017). Efficacy of (+)-Lariciresinol to control bacterial growth of *Staphylococcus aureus* and *Escherichia coli* O157. Front. Microbiol..

[119] Mehta J., Rolta R., Dev K. (2022). Role of medicinal plants from North Western Himalayas as an efflux pump inhibitor against MDR AcrAB-TolC *Salmonella enterica* serovar typhimurium: In vitro and in silico studies. J. Ethnopharmacol..

[120] Zhao X., Wang Y., Wang R., Shen J., Wang J., Li L. (2023). Lariciresinol protects rats from complete Freund’s adjuvant-induced arthritis in rats via modulation of transforming growth factor-β and nuclear factor kappa B pathway: An in vivo and in silico study. Chem. Biol. Drug Des..

[121] Saarinen N.M., Wärri A., Dings R.P., Airio M., Smeds A.I., Mäkelä S. (2008). Dietary lariciresinol attenuates mammary tumor growth and reduces blood vessel density in human MCF-7 breast cancer xenografts and carcinogen-induced mammary tumors in rats. Int. J. Cancer.

[122] Ma Z.J., Lu L., Yang J.J., Wang X.X., Su G., Wang Z.L., Chen G.H., Sun H.M., Wang M.Y., Yang Y. (2018). Lariciresinol induces apoptosis in HepG2 cells via mitochondrial-mediated apoptosis pathway. Eur. J. Pharmacol..

[123] Kezimana P., Dmitriev A.A., Kudryavtseva A.V., Romanova E.V., Melnikova N.V. (2018). Secoisolariciresinol Diglucoside of Flaxseed and Its Metabolites: Biosynthesis and Potential for Nutraceuticals. Front. Genet..

[124] Felmlee M.A., Woo G., Simko E., Krol E.S., Muir A.D., Alcorn J. (2009). Effects of the flaxseed lignans secoisolariciresinol diglucoside and its aglycone on serum and hepatic lipids in hyperlipidaemic rats. Br. J. Nutr..

[125] Zhou H., Ren J., Li Z. (2017). Antibacterial Activity and Mechanism of Pinoresinol from *Cinnamomum camphora* Leaves against Food-Related Bacteria. Food Control.

[126] Wikul A., Damsud T., Kataoka K., Phuwapraisirisan P. (2012). (+)-Pinoresinol is a putative hypoglycemic agent in defatted sesame (*Sesamum indicum*) seeds though inhibiting α-glucosidase. Bioorg. Med. Chem. Lett..

[127] Soltani M., Fotovat R., Sharifi M., Ahmadian Chashmi N., Behmanesh M. (2024). In Vitro Comparative Study on Antineoplastic Effects of Pinoresinol and Lariciresinol on Healthy Cells and Breast Cancer-Derived Human Cells. Iran. J. Med. Sci..

[128] Wei Y., Xiao L., Yingying L., Haichen W. (2022). Pinoresinol diglucoside ameliorates H/R-induced injury of cardiomyocytes by regulating miR-142-3p and HIF1AN. J. Biochem. Mol. Toxicol..

[129] Youssef F.S., Ashour M.L., El-Beshbishy H.A., Ahmed Hamza A., Singab A.N.B., Wink M. (2020). Pinoresinol-4-O-β-D-Glucopyranoside: A Lignan from Prunes (*Prunus domestica*) Attenuates Oxidative Stress, Hyperglycaemia and Hepatic Toxicity in Vitro and in Vivo. J. Pharm. Pharmacol..

[130] Zhang Y., Lei Y., Yao X., Yi J., Feng G. (2021). Pinoresinol diglucoside alleviates ischemia/reperfusion-induced brain injury by modulating neuroinflammation and oxidative stress. Chem. Biol. Drug Des..

[131] Zuo Y., Chen C., Liu F., Hu H., Dong S., Shen Q., Zeng J., Huang L., Liao X., Cao Z. (2024). Pinoresinol diglucoside mitigates dexamethasone-induced osteoporosis and chondrodysplasia in zebrafish. Toxicol. Appl. Pharmacol..

[132] Milder I.E.J., Feskens E.J.M., Arts I.C.W., de Mesquita H.B.B., Hollman P.C.H., Kromhout D. (2005). Intake of the Plant Lignans Secoisolariciresinol, Matairesinol, Lariciresinol, and Pinoresinol in Dutch Men and Women. J. Nutr..

[133] He X., Yang F., Huang X. (2021). Proceedings of Chemistry, Pharmacology, Pharmacokinetics and Synthesis of Biflavonoids. Molecules.

[134] Innocenti M., Michelozzi M., Giaccherini C., Ieri F., Vincieri F.F., Mulinacci N. (2007). Flavonoids and biflavonoids in Tuscan berries of *Juniperus communis* L.: Detection and quantitation by HPLC/DAD/ESI/MS. J. Agric. Food Chem..

[135] Yu S., Yan H., Zhang L., Shan M., Chen P., Ding A., Li S.F.Y. (2017). A Review on the Phytochemistry, Pharmacology, and Pharmacokinetics of Amentoflavone, a Naturally-Occurring Biflavonoid. Molecules.

[136] Lamer-Zarawska E. (1975). Biflavonoids in *Juniperus* species (Cupressaceae). Pol. J. Pharmacol. Pharm..

[137] Tuli H.S., Joshi H., Vashishth K., Ramniwas S., Varol M., Kumar M., Rani I., Rani V., Sak K. (2023). Chemopreventive mechanisms of amentoflavone: Recent trends and advancements. Naunyn Schmiedebergs Arch. Pharmacol..

[138] Xiong X., Tang N., Lai X., Zhang J., Wen W., Li X., Li A., Wu Y., Liu Z. (2021). Insights into amentoflavone: A natural multifunctional biflavonoid. Front. Pharmacol..

[139] Deng Z., Sheng F., Yang S.-Y., Liu Y., Zou L., Zhang L.-L. (2023). A Comprehensive Review on the Medicinal Usage of *Podocarpus* Species: Phytochemistry and Pharmacology. J. Ethnopharmacol..

[140] Ahmad S., Razaq S. (1976). New Synthesis of Biflaves of Cupressuflavone Series.

[141] Qiao Y., Sun W.-W., Wang J.-F., Zhang J.-D. (2014). Flavonoids from *Podocarpus macrophyllus* and Their Cardioprotective Activities. J. Asian Nat. Prod. Res..

[142] Jegal J., Park S.A., Chung K., Chung H.Y., Lee J., Jeong E.J., Kim K.H., Yang M.H. (2016). Tyrosinase inhibitory flavonoid from *Juniperus communis* fruits. Biosci. Biotechnol. Biochem..

[143] Zhang J., Wang Y. (2021). Bilobetin, a Novel Small Molecule Inhibitor Targeting Influenza Virus Polymerase Acidic (PA) Endonuclease Was Screened from Plant Extracts. Nat. Prod. Res..

[144] Lee H.K., Bae S., Lee J., Cha H.S., Nam M.J., Lee J., Park K., Yang Y.H., Jang K.Y., Liu K.H. (2023). Bilobetin induces apoptosis in human hepatocellular carcinoma cells via ROS level elevation and inhibition of CYP2J2. Arab. J. Chem..

[145] Islam M.T., Zihad S.M.N.K., Rahman M.S., Sifat N., Khan M.R., Uddin S.J., Rouf R. (2019). Agathisflavone: Botanical Sources, Therapeutic Promises, and Molecular Docking Study. IUBMB Life.

[146] Andrade A.W.L., Machado K.D.C., Machado K.D.C., Figueiredo D.D.R., David J.M., Islam M.T., Uddin S.J., Shilpi J.A., Costa J.P. (2018). In Vitro Antioxidant Properties of the Biflavonoid Agathisflavone. Chem. Cent. J..

[147] Lin Y.-M., Zembower D.E., Flavin M.T., Schure R.M., Anderson H.M., Korba B.E., Chen F.-C. (1997). Robustaflavone, a Naturally Occurring Biflavanoid, Is a Potent Non-Nucleoside Inhibitor of Hepatitis B Virus Replication in Vitro. Bioorg. Med. Chem. Lett..

[148] Hiermann A., Kompek A., Reiner J., Auer H., Schubert-Zsilavecz M. (1996). Investigation of flavonoid pattern in fruits of *Juniperus communis* L. Sci. Pharm..

[149] Lee C.C., Hsiao C.Y., Lee S.C., Huang X.F., Chang K.F., Lee M.S., Hsieh M.C., Tsai N.M. (2020). Suppression of Oral Cancer by Induction of Cell Cycle Arrest and Apoptosis Using *Juniperus communis* Extract. Biosci. Rep..

[150] Reddy P.J., Sinha S., Ray S., Sathe G.J., Chatterjee A., Prasad T.S., Dhali S., Srikanth R., Panda D., Srivastava S. (2015). Comprehensive analysis of temporal alterations in cellular proteome of Bacillus subtilis under curcumin treatment. PLoS ONE.

[151] Xu Z., Krajewski S., Weindl T., Loeffler R., Li P., Han X., Geis-Gerstorfer J., Wendel H.P., Scheideler L., Rupp F. (2020). Application of Totarol as natural antibacterial coating on dental implants for prevention of peri-implantitis. Mater. Sci. Eng. C.

[152] Scariot D.B., Volpato H., Fernandes N.D., Soares E.F., Ueda-Nakamura T., Dias-Filho B.P., Din Z.U., Rodrigues-Filho E., Rubira A.F., Borges O. (2019). Activity and cell-death pathway in *Leishmania infantum* induced by sugiol: Vectorization using yeast cell wall particles obtained from *Saccharomyces cerevisiae*. Front. Cell. Infect. Microbiol..

[153] Jung S.N., Shin D.S., Kim H.N., Jeon Y.J., Yun J., Lee Y.J., Kang J.S., Han D.C., Kwon B.M. (2015). Sugiol inhibits STAT3 activity via regulation of transketolase and ROS-mediated ERK activation in DU145 prostate carcinoma cells. Biochem. Pharmacol..

[154] Bakhsh T., Abuzahrah S.S., Qahl S.H., Akela M.A., Rather I.A. (2023). Sugiol masters apoptotic precision to halt gastric cancer cell proliferation. Pharmaceuticals.

[155] Bajpai V.K., Sharma A., Chul Kang S., Baek K.-H., Chul Kang S. (2014). Antioxidant, lipid peroxidation inhibition, and free radical scavenging efficacy of a diterpenoid compound sugiol isolated from *Metasequoia glyptostroboides*. Asian Pac. J. Trop. Med..

[156] Karalija E., Šamec D. (2023). Amentoflavone: Structure, resources, bioactivity, and pharmacology. Handbook of Dietary Flavonoids.

[157] Youn K.W., Lee S., Kim J.H., Park Y.I., So J., Kim C., Cho C.W., Park J. (2024). Amentoflavone from *Selaginella tamariscina* inhibits SARS-CoV-2 RNA-dependent RNA polymerase. Heliyon.

[158] Al-Sayed E., Gad H.A., El-Shazly M., Abdel-Daim M.M., Nasser Singab A. (2018). Anti-inflammatory and analgesic activities of cupressuflavone from *Cupressus macrocarpa*: Impact on pro-inflammatory mediators. Drug Dev. Res..

[159] Al-Sayed E., Ke T.Y., Hwang T.L., Chen S.R., Korinek M., Chen S.L., Cheng Y.B. (2020). Cytotoxic and anti-inflammatory effects of lignans and diterpenes from *Cupressus macrocarpa*. Bioorg. Med. Chem. Lett..

[160] Kim C.E., Le D.D., Lee M. (2021). Diterpenoids isolated from *Podocarpus macrophyllus* inhibited the inflammatory mediators in LPS-induced HT-29 and RAW 264.7 cells. Molecules.

[161] Mohamed N.Z., Shaaban L., Safan S., El-Sayed A.S.A. (2023). Phytochemical and metabolic profiling of the different *Podocarpus* species in Egypt: Potential antimicrobial and antiproliferative activities. Heliyon.

[162] Chaves O.A., Lima C.R., Fintelman-Rodrigues N., Sacramento C.Q., De Freitas C.S., Vazquez L., Temerozo J.R., Rocha M.E., Dias S.S., Carels N. (2022). Agathisflavone, a natural biflavonoid that inhibits SARS-CoV-2 replication by targeting its proteases. Int. J. Biol. Macromol..

[163] Jo A., Yoo H.J., Lee M. (2019). Robustaflavone isolated from *Nandina domestica* using bioactivity-guided fractionation downregulates inflammatory mediators. Molecules.

[164] Sim W.K., Park J.H., Kim K.Y., Chung I.S. (2020). Robustaflavone induces G0/G1 cell cycle arrest and apoptosis in human umbilical vein endothelial cells and exhibits anti-angiogenic effects in vivo. Sci. Rep..

[165] El Jemli M., Kamal R., Marmouzi I., Zerrouki A., Cherrah Y., Alaoui K. (2016). Radical-scavenging activity and ferric reducing ability of *Juniperus thurifera* (L.), *J. oxycedrus* (L.), *J. phoenicea* (L.) and *Tetraclinis articulata* (L.). Adv. Pharmacol. Sci..

[166] Elmastaş M., Gülçin I., Beydemir Ş., Küfrevioğlu Ö.I., Aboul-Enein H.Y. (2006). A study on the in vitro antioxidant activity of juniper (*Juniperus communis* L.) fruit extracts. Anal. Lett..

[167] Hiller K., Löw D., Wichtl M. (2009). Juniperi Pseudo-Fructus. Teedrogen und Phytopharmaka.

[168] Zheljazkov V.D., Semerdjieva I.B., Dincheva I., Kacaniova M., Astatkie T., Radoukova T., Schlegel V. (2017). Antimicrobial and antioxidant activity of *Juniper galbuli* essential oil constituents eluted at different times. Ind. Crop. Prod..

[169] Bojor O. (2003). Ghidul Plantelor Medicinale şi Aromatice de la A la Z (Guide of Medicinal and Aromatic Plants from A to Z).

[170] Muftah H., Ozçelik B., Oyardı O., Kutluk İ., Orhan N. (2020). A comparative evaluation of *Juniperus* species with antimicrobial magistrals. Pharm. Sci..

[171] Kim D.S., Lee H.J., Jeon Y.D., Han Y.H., Kee J.Y., Kim H.J., Shin H.J., Kang J., Lee B.S., Kim S.H. (2015). Alpha-pinene exhibits anti-inflammatory activity through the suppression of MAPKs and the NF-κB pathway in mouse peritoneal macrophages. Am. J. Chin. Med..

[172] Darwish R.S., Hammoda H.M., Ghareeb D.A., Abdelhamid A.S.A., Bellah El Naggar E.M., Harraz F.M., Shawky E. (2020). Efficacy-directed discrimination of the essential oils of three *Juniperus* species based on their in-vitro antimicrobial and anti-inflammatory activities. J. Ethnopharmacol..

[173] Lin T.-C., Lu C.-W., Chang K.-F., Lee C.-J. (2022). *Juniperus communis* extract ameliorates lipopolysaccharide-induced acute kidney injury through the adenosine monophosphate–activated protein kinase pathway. Food Sci. Nutr..

[174] Tsai W.C., Tsai N.M., Chang K.F., Wang J.C. (2018). *Juniperus communis* extract exerts antitumor effects in human glioblastomas through blood-brain barrier. Cell. Physiol. Biochem..

[175] Lai W.L., Lee S.C., Chang K.F., Huang X.F., Li C.Y., Lee C.J., Wu C.Y., Hsu H.J., Tsai N.M. (2021). *Juniperus communis* extract induces cell cycle arrest and apoptosis of colorectal adenocarcinoma in vitro and in vivo. Braz. J. Med. Biol. Res..

[176] Li C.Y., Lee S.C., Lai W.L., Chang K.F., Huang X.F., Hung P.Y., Lee C.P., Hsieh M.C., Tsai N.M. (2020). Cell cycle arrest and apoptosis induction by *Juniperus communis* extract in esophageal squamous cell carcinoma through activation of p53-induced apoptosis pathway. Food Sci. Nutr..

[177] Abdelghany T.M., Hassan M.M., El-Naggar M.A. (2020). GC/MS analysis of *Juniperus procera* extract and its activity with silver nanoparticles against *Aspergillus flavus* growth and aflatoxins production. Biotechnol. Rep..

[178] Gad El-Rab S.M.F., Halawani E.M., Alzahrani S.S.S. (2021). Biosynthesis of silver nano-drug using *Juniperus excelsa* and its synergistic antibacterial activity against multidrug-resistant bacteria for wound dressing applications. 3 Biotech.

[179] Halawani E.M.S., Alzahrani S.S.S., Gad El-Rab S.M.F. (2023). Biosynthesis strategy of gold nanoparticles and biofabrication of a novel amoxicillin gold nanodrug to overcome the resistance of multidrug-resistant bacterial pathogens MRSA and *E. coli*. Biomimetics.

